# Herbal medicines against avian infectious bronchitis virus: shared host-directed and multi-stage antiviral pathways

**DOI:** 10.3389/fvets.2026.1812050

**Published:** 2026-08-24

**Authors:** Chenyuan Zhang, Yun Zheng, Chunxiao Jiang, Yijie Zhang

**Affiliations:** Department of Functional Sciences, School of Medicine, Yangtze University, Jingzhou, Hubei, China

**Keywords:** avian infectious bronchitis virus, host-directed antiviral mechanisms, immunomodulation, poultry health, traditional Chinese medicine

## Abstract

Avian infectious bronchitis virus (IBV) is a highly contagious and genetically diverse coronavirus that continues to pose a major threat to poultry production worldwide. Although vaccination remains the primary control strategy, the frequent emergence of viral variants and incomplete cross-protection among antigenically distinct strains necessitate complementary approaches for effective IBV management. Accumulating evidence suggests that traditional Chinese medicine (TCM) and herbal-derived compounds may provide protective benefits against IBV infection. Although certain herbal compounds directly target viral particles or life-cycle stages, many protective effects appear to involve host-directed regulatory mechanisms. Recurrent mechanisms include modulation of innate immune responses, particularly type I interferon signaling, together with regulation of inflammation, oxidative stress, apoptosis, autophagy, and stress-adaptive processes such as stress granule formation. In this narrative review, we searched PubMed, Web of Science, Scopus, Google Scholar, and major Chinese databases for studies related to IBV, herbal medicines, natural compounds, antiviral activity, and host-directed mechanisms. Direct IBV-specific evidence is available for only a subset of the interventions reviewed; findings from SARS-CoV, SARS-CoV-2, other non-IBV systems, and in silico analyses are treated as indirect and hypothesis-generating, and interventions supported solely by such findings are presented as candidates for IBV-specific validation rather than as established anti-IBV agents. We synthesize *in vitro* and *in vivo* evidence to identify recurrent host-response pathways and shared regulatory hubs, and propose a multi-target, multi-stage framework for the adjunctive use of herbal interventions in IBV control.

## 1 Introduction

Avian infectious bronchitis virus (IBV) is a highly contagious avian coronavirus and a major cause of respiratory and systemic disease in chickens. Taxonomically, IBV belongs to the genus Gammacoronavirus and is an enveloped, non-segmented, single-stranded positive-sense RNA virus with a genome of approximately 27.6 kb. Since its first isolation in the United States in 1931, numerous antigenically and genetically distinct IBV variants have been identified worldwide (1–5). Despite the extensive use of vaccination programs, IBV remains a persistent and economically important threat to poultry production. This continuing burden is largely driven by the high genetic variability of IBV, which promotes the emergence of novel genotypes and lineages and may also generate antigenically distinct variants, thereby limiting cross-protection among strains. In addition, the broad tissue tropism of IBV, involving the respiratory tract, kidneys, reproductive system, and gastrointestinal tract, contributes to diverse clinical manifestations and long-term production losses (2–5).

In the field, this biological diversity is reflected in the wide geographic distribution and frequent replacement of circulating IBV lineages. Continuous mutation, recombination, and antigenic drift, particularly within the spike S1 gene, contribute to the emergence and coexistence of multiple genotypes and lineages. Recent molecular surveillance studies have revealed marked regional differences in dominant IBV lineages, with QX-like/GI-19, 4/91-like/GI-13, and other genotype I lineages continuing to circulate in many poultry-producing areas (6, 7). These observations help explain why IBV outbreaks may still occur in vaccinated flocks and why broad, durable protection remains difficult to achieve with vaccination alone.

The clinical impact of IBV reflects not only viral replication but also tissue tropism and dysregulated host responses. IBV primarily infects epithelial cells of the upper respiratory tract, leading to tracheal epithelial damage, ciliostasis, mucus accumulation, and respiratory signs. Depending on the viral strain, infection may extend to extra-respiratory tissues, including the kidneys, oviduct, gastrointestinal tract, and immune organs, resulting in nephritis, reduced egg production and egg quality, impaired growth performance, and increased mortality (8). At the cellular and molecular levels, IBV infection is accompanied by excessive inflammation, oxidative imbalance, and alterations in host cell-fate regulation, which collectively contribute to tissue injury and disease progression. Therefore, IBV pathogenesis involves not only viral replication but also complex interactions between viral factors and host responses.

Current IBV control relies primarily on live attenuated and inactivated vaccines. However, vaccine-induced protection is often strain-dependent and may decline over time under field conditions, particularly when circulating field variants are antigenically mismatched with vaccine strains. Such antigenic mismatches can substantially reduce protective efficacy and help explain the continued occurrence of IBV outbreaks in vaccinated flocks. Moreover, vaccines are mainly designed to induce viral neutralization, whereas several important contributors to IBV-associated pathology, including excessive inflammation, oxidative stress, and immune dysregulation, are not fully addressed by vaccination alone.

Against this background, herbal medicines have garnered increasing attention as complementary approaches for IBV control (9–11). Unlike antiviral strategies that focus mainly on viral components, many herbal-derived compounds are being investigated for their ability to modulate host responses, including enhancement of innate antiviral immunity, restoration of immune homeostasis, and attenuation of virus-induced inflammatory injury. Because herbal interventions often contain multiple bioactive constituents, they may influence several interconnected processes during infection, such as antiviral signaling, oxidative stress, autophagy, and apoptosis, thereby reshaping host–virus interactions rather than acting through a single molecular target (12).

Although experimental studies have reported antiviral and immunomodulatory activities of individual herbs, bioactive compounds, and compound formulations against IBV, the available evidence remains fragmented. Most studies focus on specific herbs, isolated constituents, or individual prescriptions, making it difficult to determine whether these interventions act through unrelated effects or share overlapping host-response pathways. In particular, the host-directed mechanisms that may connect diverse herbal interventions have not been sufficiently synthesized.

Therefore, this review provides a mechanism-oriented synthesis of herbal medicines against IBV, with a particular focus on host-directed antiviral pathways. By integrating evidence from *in vitro* studies, *in vivo* models, and network pharmacology analyses, we highlight a multi-stage and multi-target regulatory framework through which herbal medicines may modulate viral replication, immune responses, inflammation, and tissue injury.

To ensure methodological transparency, we searched PubMed, Web of Science, Scopus, Google Scholar, CNKI, and Wanfang Data from database inception to June 1, 2026, without language restrictions. A representative PubMed search strategy combined MeSH terms and free-text keywords: (“avian infectious bronchitis virus” OR “IBV”) AND (“traditional Chinese medicine” OR “herbal medicine” OR “natural compound” OR “polysaccharide” OR “flavonoid” OR “compound formulation”) AND (“antiviral” OR “immunomodulation” OR “interferon” OR “inflammation” OR “oxidative stress” OR “apoptosis” OR “autophagy” OR “stress granule”); this strategy was adapted for other databases. Duplicates were removed using EndNote X20, followed by manual verification. Two independent reviewers (C. Z. and Y. Z.) screened titles and abstracts and then assessed full texts against the eligibility criteria; disagreements were resolved by discussion or consultation with a third reviewer (C. J.). Studies were included if they reported herbal or natural interventions relevant to IBV infection, IBV vaccination, coronavirus antiviral mechanisms, or host-directed antiviral pathways. Studies were excluded if they lacked mechanistic relevance to IBV infection or vaccination, did not report original experimental data (e.g., reviews, commentaries, or conference abstracts), or provided insufficient methodological detail for result interpretation. Review articles were excluded from the formal evidence set because they did not report original data. However, selected review articles were cited separately to provide general background, methodological context, or broader pharmacological interpretation and were not counted among the included studies. After screening and eligibility assessment, 46 original studies were included in the formal evidence set. The manuscript cited 123 publications in total; the remaining 77 publications comprised review articles and sources used only for general background or methodological context and were not counted as included studies. Studies on non-IBV coronaviruses or original studies based solely on computational analyses were not excluded but were categorized separately as indirect or hypothesis-generating evidence. No meta-analysis was performed; instead, studies were categorized by experimental model (cell culture, chicken embryo, live chicken, other coronavirus, or in silico), and evidence from non-IBV systems was treated as indirect and hypothesis-generating. Direct IBV-specific evidence was prioritized when interpreting antiviral efficacy, whereas findings from other coronavirus models or computational analyses were considered supportive mechanistic evidence requiring further IBV-specific validation.

## 2 Research on single herbal medicines and their extracts against IBV

In recent years, a growing number of studies have investigated the anti-IBV potential of single herbal medicines, herbal extracts, and their bioactive constituents (11, 13–24). These studies have covered several major classes of natural compounds, including polysaccharides such as Astragalus polysaccharide and Radix Isatidis polysaccharide (14, 15), flavonoids and phenolic compounds such as baicalin, phillygenin, forsythoside A, elderberry extract, hypericin, and myricetin (16–22, 25, 26), as well as alkaloids and other plant-derived constituents or extracts (23, 24). Their antiviral activity has been evaluated in experimental models ranging from *in vitro* cell-based assays to chicken embryo and live bird studies (14, 15, 23, 24). Although the depth of mechanistic evidence varies among different agents, these findings provide an important basis for understanding how individual herbal medicines may interfere with IBV infection or modulate host responses (14–18).

### 2.1 Polysaccharides from herbal medicines

Polysaccharides are important bioactive macromolecules in many herbal medicines and are widely recognized for their immunomodulatory properties. In traditional Chinese medicine, polysaccharide-rich herbs are often associated with immune-enhancing effects, and modern pharmacological studies increasingly support their roles in regulating innate immunity, inflammation, oxidative stress, apoptosis, and different stages of viral infection (27, 28). In the context of IBV, several herbal polysaccharides have shown antiviral or immunomodulatory activity, making them a relevant group of natural compounds for host-directed intervention.

#### 2.1.1 Astragalus polysaccharide

APS is one of the primary active components extracted from *Astragalus mongholicus*, a classic tonic herb in traditional Chinese medicine. As a well-known immunomodulatory polysaccharide, APS exhibits antiviral and immune-regulatory activities in various experimental systems (29). Although broader pharmacological studies have linked APS to inflammatory and oxidative pathways, these mechanisms remain indirect with respect to IBV infection. IBV-specific studies have therefore mainly focused on its antiviral activity and regulation of host immune responses.

In IBV-specific studies, APS appears to play a dual role by suppressing viral replication and modulating inflammatory responses. *In vitro* experiments have shown that APS dose-dependently inhibits IBV replication in chicken embryo kidney (CEK) cells and reduces viral titers (14). Meanwhile, APS downregulates the mRNA expression of pro-inflammatory cytokines, including IL-1β, IL-6, IL-8, and TNF-*α*, in infected cells, which may help limit excessive cytokine production and reduce virus-induced inflammatory injury (14).

Furthermore, APS can serve as an effective immune adjuvant. In an immunization trial with an IBV DNA vaccine, oral administration of APS at doses of 50 and 100 mg/kg significantly increased specific antibody titers, enhanced lymphocyte proliferation, and upregulated the splenic mRNA expression of IL-1β, IL-2, IL-8, and TNF-*α* in chickens. These changes augmented both cellular and humoral immune responses (30). These multifaceted actions support its potential as a multi-target adjunct strategy for the prevention and control of infectious bronchitis (14, 31) (Table 1).

**Table 1:** Effects of APS and RIP on IBV infection and immune-related responses.

Traditional medicine/Component	Experimental model/Cell	Tested IBV strain	Mechanism of action	Reference
APS	CEK cells	IBV-M41 strain	IBV N protein↓; IL-1β↓; IL-6↓; IL-8↓; TNF-α↓	(14)
	Chicken	IBV-M41 strain	IL-1β↑^i^; IL-2↑; IL-8↑; TNF-α↑	(30)
	Chicken	Not clearly specified	IgG↑; IgM↑; IgA↑; MDA↓ This is indirect immunomodulatory evidence and does not demonstrate anti-IBV efficacy	(31)
RIP	CEK cells	QX-type IBV	IL-6↓; IL-8↓; IL-1β↓; MDA5↑; MAVS↑; TLR3↑; TRIF↑; IRF7↑; IFN-β↑	(15)

↑ indicates upregulation and ↓ indicates downregulation. ^i^Immunization adjuvant context; cytokine upregulation reflects enhanced vaccine response.

#### 2.1.2 Polysaccharides from *Radix isatidis*

Radix Isatidis (Banlangen) is a well-known antiviral medicinal herb that was officially recommended in China during the Severe Acute Respiratory Syndrome (SARS) outbreak (11). Its polysaccharide fraction, Radix Isatidis polysaccharide (RIP), has also been shown to demonstrate potent antiviral activity against IBV. In particular, Xiang et al. reported that RIP effectively inhibited the nephropathogenic IBV QX strain (15). *In vivo* chick models, RIP administration significantly alleviated IBV-induced renal injury, while *in vitro* studies demonstrated a marked reduction in viral replication in chicken embryo kidney (CEK) cells.

**Figure 1:**
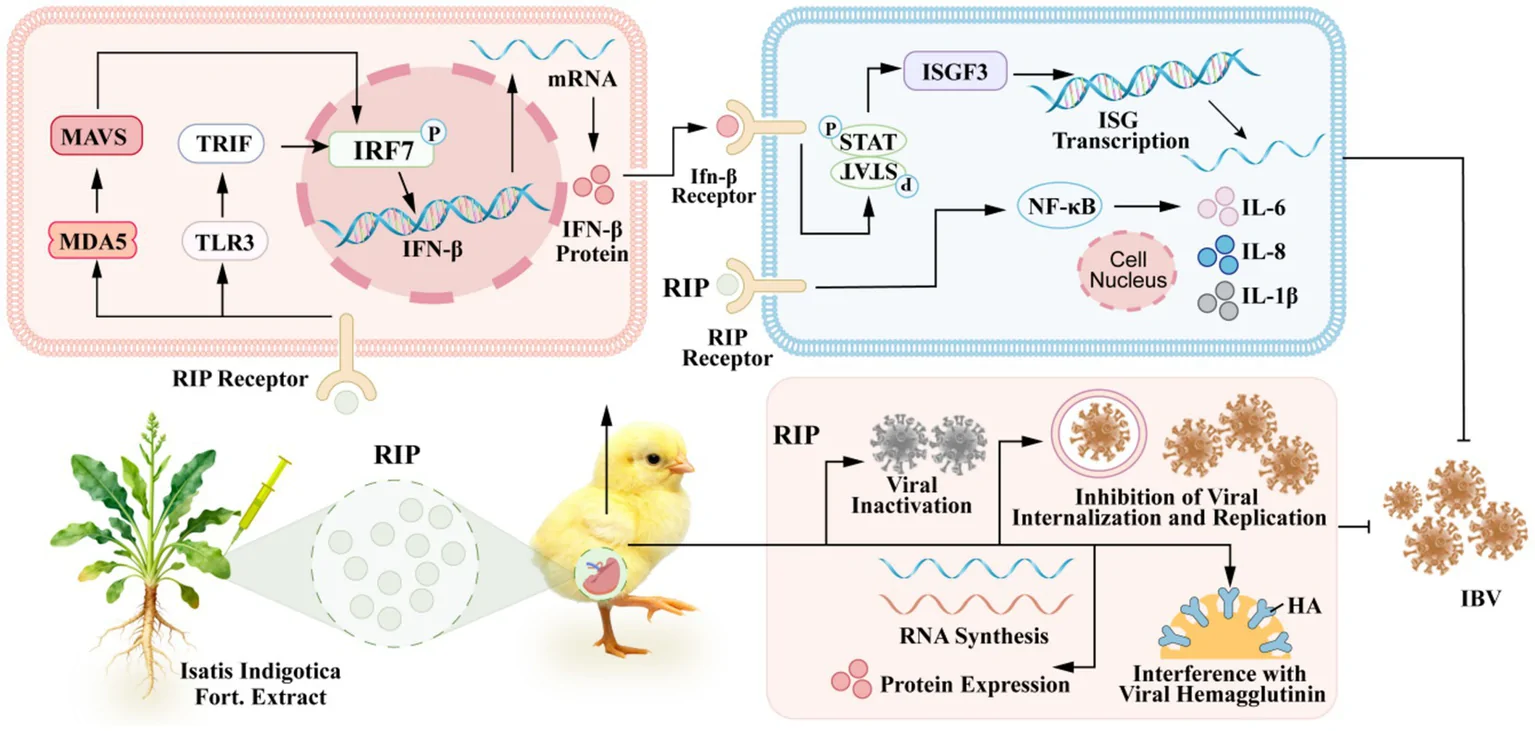
Schematic illustration of the antiviral and immunomodulatory effects of Radix Isatidis polysaccharide (RIP) against infectious bronchitis virus (IBV). RIP is associated with activation of innate immune signaling, including upregulation of MDA5 and TLR3 and downstream IRF7-related induction of type I interferon (IFN-*β*), thereby contributing to an antiviral state. RIP is also associated with reduced expression of pro-inflammatory cytokines and attenuation of inflammatory responses in IBV-infected systems. In addition, RIP has been reported to inhibit IBV internalization and replication. Potential immune-enhancing effects of RIP in a vaccine-adjuvant context are not presented here as established IBV-specific mechanisms, because the supporting evidence is indirect and was not demonstrated in an IBV-vaccination model.

Mechanistically, the anti-IBV activity of RIP is associated with activation of host innate immune signaling. RIP upregulates pattern-recognition receptors including MDA5 and TLR3, together with downstream IRF7, thereby promoting type I interferon (IFN-*β*) signaling and establishment of an antiviral state (15). Concurrently, RIP suppresses NF-κB signaling and reduces the production of pro-inflammatory cytokines, including IL-6, IL-8, and IL-1β (15). Its potential as a vaccine-associated immune enhancer requires further validation using standardized preparations and well-controlled poultry models (15, 32) (Figure 1; Table 1).

#### 2.1.3 Other polysaccharides with coronavirus-relevant antiviral potential

Apart from APS and RIP, which have been directly evaluated in IBV infection models, the anti-IBV activity of most other herbal or natural polysaccharides remains insufficiently validated. Nevertheless, studies on other coronaviruses, especially SARS-CoV and SARS-CoV-2, suggest that structurally diverse polysaccharides may interfere with coronavirus infection through both virus-targeted and host-directed mechanisms (33–35). Therefore, these polysaccharides should be viewed as candidates for future anti-IBV screening rather than as agents with established anti-IBV efficacy.

Several polysaccharides have shown coronavirus-relevant antiviral activities by interfering with viral attachment and entry. Sulfated polysaccharides, such as carrageenan, fucoidan, sea cucumber sulfated polysaccharide, and heparin-like polysaccharides, can mimic host cell-surface glycosaminoglycans and competitively interact with viral envelope glycoproteins or host attachment factors, thereby reducing coronavirus binding and entry into susceptible cells (36). For example, marine sulfated polysaccharides, including sea cucumber sulfated polysaccharide, fucoidan, and iota-carrageenan, have been reported to inhibit SARS-CoV-2 infection *in vitro*, and the inhibitory activity was associated with interference with spike glycoprotein-mediated viral entry (37). Since the spike glycoprotein is also essential for IBV attachment and entry, these findings provide a useful mechanistic reference for screening entry-blocking polysaccharides against IBV. However, because the receptor usage, tissue tropism, and spike structure of IBV differ from those of mammalian coronaviruses, their anti-IBV activity must be confirmed using IBV-specific cell, embryo, and chicken infection models.

In addition to entry interference, many herbal, fungal, and marine polysaccharides exhibit broad immunomodulatory, anti-inflammatory, and antioxidant activities that may be relevant to IBV-associated disease (38). For example, polysaccharides derived from Echinacea, ginseng, licorice, *Lycium barbarum*, Cordyceps species, and other medicinal sources have been reported to regulate macrophage activation, natural killer cell activity, cytokine secretion, and Toll-like receptor–mediated innate immune signaling in different experimental settings (39). These host-directed activities are theoretically relevant to IBV infection, because IBV pathogenesis is closely associated with innate immune activation, inflammatory injury, oxidative stress, and epithelial damage. In particular, polysaccharide-mediated regulation of pattern-recognition receptor signaling and interferon-related responses may help establish an antiviral state, while anti-inflammatory and antioxidant effects may reduce virus-induced tissue injury.

Despite these promising indications, current evidence for these polysaccharides in IBV infection remains largely indirect. Most studies have been performed in non-IBV viral models or general immunomodulatory systems, and the effective concentrations, pharmacokinetics, tissue distribution, and safety profiles in poultry remain unclear. Therefore, future studies should prioritize systematic evaluation of these candidate polysaccharides in IBV-specific models. Such studies should include *in vitro* antiviral screening in chicken embryo kidney cells or other IBV-permissive systems, chicken embryo and live bird challenge assays, viral load quantification, histopathological examination, and mechanistic validation of key pathways such as S protein-mediated entry, PRR–IFN signaling, NF-κB-mediated inflammation, oxidative stress, and epithelial cell death. This evidence hierarchy will help distinguish true anti-IBV polysaccharide candidates from broadly immunostimulatory agents and support the rational development of safe polysaccharide-based adjuncts for IBV prevention and control.

### 2.2 Flavonoids and phenolic compounds

Flavonoids and phenolic compounds, a class of widely distributed secondary metabolites in traditional Chinese medicine, are characterized by diverse pharmacological activities, including notable anti-inflammatory (40) and antiviral (25) effects. In the context of IBV infection, multiple herbal medicines and extracts enriched in these compounds have demonstrated promising antiviral efficacy, attracting increasing research interest.

#### 2.2.1 Baicalin

Baicalin, a representative flavonoid constituent of Scutellaria baicalensis Georgi, is one of the most extensively studied herbal compounds in anti-IBV research (11, 16, 17, 26). *In vitro* studies have shown that baicalin can directly inactivate IBV particles, exhibiting virucidal activity, whereas prophylactic treatment shows limited efficacy (16).

Additionally, in cell-based assays, baicalin inhibits multiple stages of the viral life cycle, including viral adsorption, entry, replication, and release. In IBV-infected Vero cells, baicalin treatment significantly reduced both mRNA and protein levels of the viral nucleocapsid (N) protein in a dose-dependent manner. This intracellular antiviral effect is mediated, at least in part, by activation of the host PKR/eIF2α pathway and subsequent induction of stress granule (SG) formation, leading to translational arrest of viral proteins (16).

**Figure 2:**
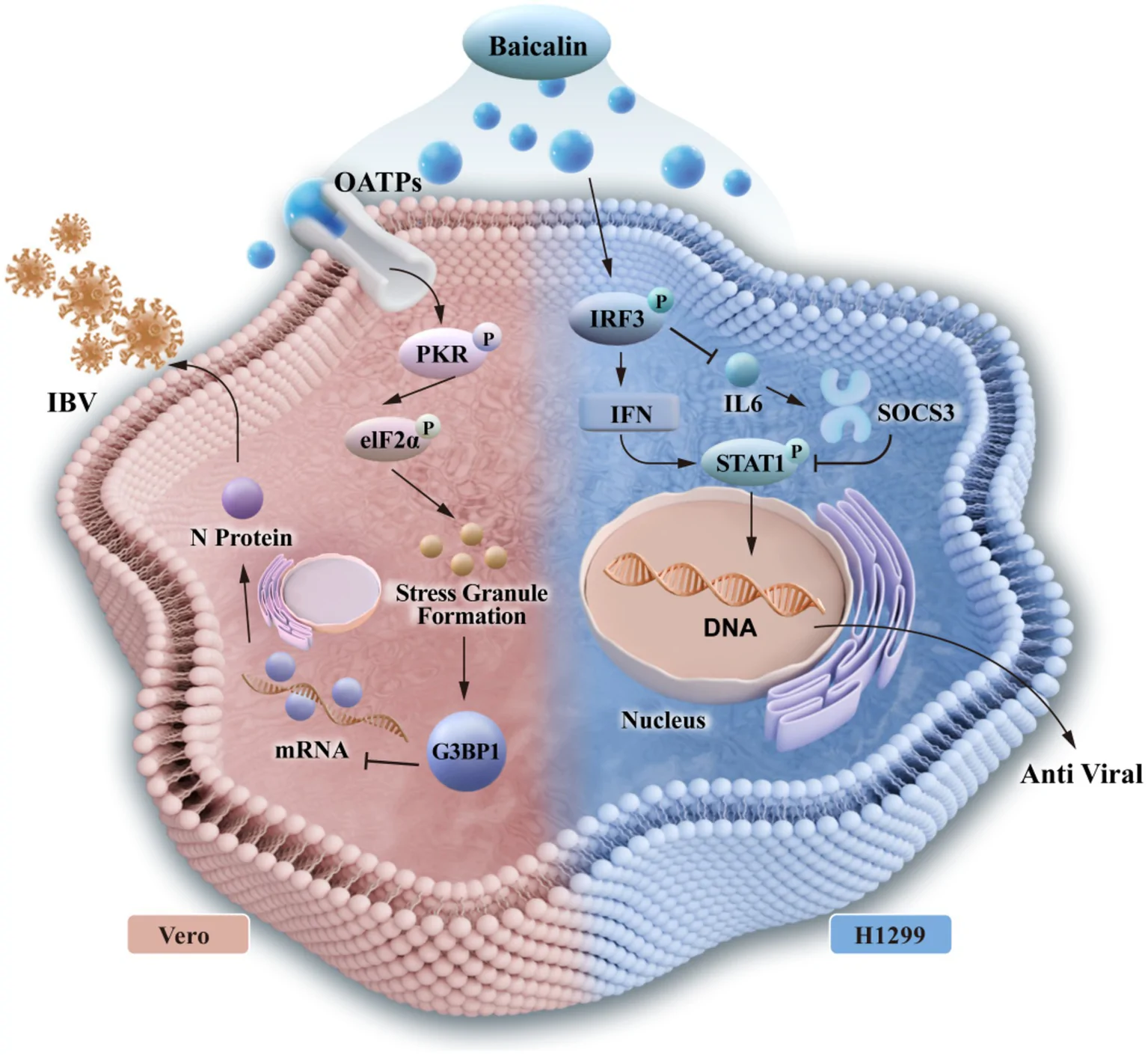
Antiviral mechanisms of baicalin against infectious bronchitis virus (IBV). Baicalin inhibits IBV infection by suppressing multiple stages of the viral life cycle and activating host antiviral responses. In infected cells, baicalin induces PKR/eIF2*α* phosphorylation and stress granule formation, leading to reduced viral mRNA translation and decreased nucleocapsid (N) protein expression. Concurrently, baicalin enhances type I interferon signaling through IRF3 and STAT1 activation while inhibiting negative regulators, thereby establishing an antiviral state.

In addition to SG-mediated translational suppression, baicalin also enhances host antiviral innate immunity. In IBV-infected H1299 cells, baicalin treatment administered either before or after viral adsorption significantly inhibited viral replication in a concentration-dependent manner. This effect was associated with activation of the type I interferon (IFN) signaling cascade, as evidenced by increased expression of p-IRF3, IFN-*β*, and p-STAT1, along with suppression of the negative regulators SOCS3 and IL-6 within the JAK–STAT pathway. Enhanced nuclear translocation of p-STAT1 and increased sensitivity of IBV to exogenous IFN-*α* were also observed. Notably, STAT1 knockdown abrogated the antiviral effect of baicalin, confirming a central role for STAT1-dependent type I IFN signaling in baicalin-mediated inhibition of IBV replication (17) (Figure 2; Table 2).

**Table 2:** Effects of baicalin, phillygenin and forsythoside A on IBV infection and host immune responses.

Traditional medicine/component	Original source and chemical structure information	Experimental model/cell	Tested IBV strain	Mechanism of action	Reference
Baicalin	C₂₁H₁₈O₁₁; PubChem CID: 64982.	Chicken	Nephropathogenic infectious bronchitis virus (NIBV)	iNOS↑; TNF-α↑; IL-1β↑; IL-6↑; ARG2↓; IL-4↓; IL-10↓; PPARG↓	(26)
		Vero cells	IBV Beaudette strain	p-PKR↑; p-eIF2α↑; SG↑; G3BP1↑; IBV N protein↓	(16)
		H1299 cells	IBV-p65 strain	p-IRF3↑; IFN-β↑; p-STAT1↑; IL-6↓; SOCS3↓	(17)
Phillygenin	C₂₁H₂₄O₆; PubChem CID: 4166098.	Vero cells	IBV Beaudette strain	IBV N protein↓; p-eIF2α↑; G3BP1↑; p-PKR↑; SG↑	(18)
Forsythoside A	C₂₉H₃₆O₁₅; PubChem CID: 5281773.	BHK cells	IBV Beaudette strain/IBV-p65 strain	IBV-induced cytopathic effects↓; Beclin-1↓; LC3-II↓; PI3K/Akt/NF-κB signaling↓; Caspase-3↓; changes in Bax/BCL-2-associated signaling. Complete autophagic flux was not established	(25)

↑ indicates upregulation and ↓ indicates downregulation. Changes in LC3-II and Beclin-1 represent static autophagy-associated marker changes and should not be interpreted independently as evidence that complete autophagic flux is induced or inhibited.

#### 2.2.2 Phillygenin and forsythoside a

Phillygenin, a lignan constituent of *Forsythia suspensa*, has demonstrated anti-IBV activity in infected Vero cells. Phillygenin treatment inhibits viral replication and induces a PKR/eIF2α-dependent antiviral response, with G3BP1 involvement confirmed by knockdown experiments (18). The detailed role of stress-granule-mediated translational control is discussed in the host-directed antiviral mechanisms section.

In parallel, forsythoside A, a representative phenylethanoid glycoside from *F. suspensa*, has also demonstrated significant anti-IBV efficacy. Forsythoside A effectively alleviates IBV-induced cytopathic effects and cell death by modulating the PI3K/Akt/NF-κB signaling axis, with accompanying changes in autophagy- and apoptosis-associated markers (25, 41). Because static changes in LC3-II or Beclin-1 do not establish lysosomal degradation or complete autophagic flux, these findings should be interpreted as modulation of autophagy-associated responses rather than definitive suppression of autophagy. In addition to these host-directed effects, forsythoside A can directly bind to the IBV S1 subunit, blocking viral entry and exerting a direct anti-infective action (42).

**Figure 3:**
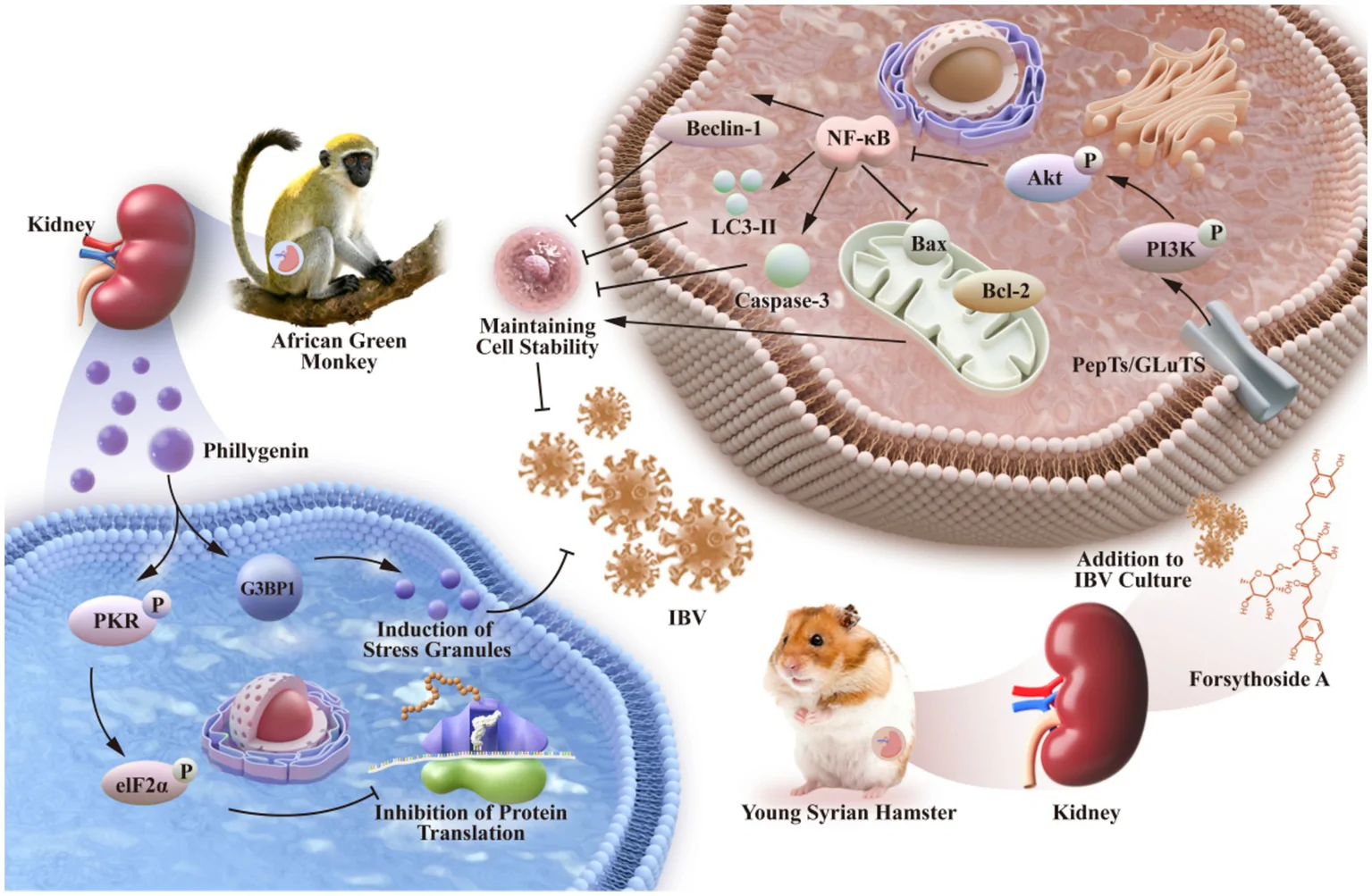
Antiviral mechanisms of Phillygenin and Forsythoside A against infectious bronchitis virus (IBV). Phillygenin suppresses IBV replication by activating the host PKR/eIF2α/G3BP1 stress-granule response, resulting in inhibition of viral protein translation. Forsythoside A attenuates IBV-induced cytopathic effects and is associated with changes in PI3K/Akt/NF-κB signaling and autophagy- and apoptosis-associated markers; complete autophagic flux was not established. It also directly interferes with viral entry by binding to the IBV S1 subunit.

Together, these findings suggest that different constituents of *F. suspensa* act through complementary mechanisms, including SG-mediated translational suppression, regulation of inflammation- and cell fate-related signaling pathways, and direct interference with viral entry. This mechanistic diversity may partly explain the frequent inclusion of *F. suspensa* in compound formulations used for respiratory viral infections, including avian infectious bronchitis (Figure 3; Table 2).

#### 2.2.3 Elderberry (*Sambucus nigra*)

Elderberry (*Sambucus nigra*), although not a traditional Chinese medicinal herb, is rich in polyphenolic compounds, including anthocyanins and flavonoids, which confer potent antioxidant and antiviral properties (43). Its activity against IBV has been experimentally demonstrated (44). Notably, Chen et al. reported that treatment with an ethanol extract of *S. nigra* reduced IBV titers by 4–6 log₁₀, indicating strong virucidal efficacy (19).

Mechanistic analyses suggest that the antiviral activity of elderberry extract is primarily mediated through direct disruption of the viral envelope at an early stage of infection. Transmission electron microscopy revealed pronounced envelope damage and membrane vesicle formation in IBV particles following extract treatment, supporting a direct viral inactivation mechanism rather than host-mediated antiviral regulation (19). These findings suggest that polyphenol-rich plant extracts may serve as natural viral inactivating agents, with potential applications in reducing viral contamination or transmission risk. However, their practical use in poultry settings will require further evaluation of formulation stability, safety, and delivery methods (45).

### 2.3 Alkaloids and other constituents

In addition to polysaccharides and flavonoids, alkaloids and other bioactive constituents from selected medicinal plants have also been reported to exhibit anti-IBV activity, further expanding the spectrum of natural compounds with therapeutic potential against IBV (11, 46).

#### 2.3.1 Houttuynia cordata

*Houttuynia cordata* (HC), also known as Zhe’ergen, is a medicinal and edible plant traditionally used in Chinese medicine for respiratory and inflammatory disorders. In modern pharmacological terms, its reported activities are mainly associated with anti-inflammatory, antimicrobial, antiviral, and tissue-protective effects. Crude extracts of the whole plant contain multiple bioactive constituents, including flavonoids (e.g., quercetin) and polysulfides (47, 48), and have demonstrated pronounced anti-IBV activity in both *in vitro* and *in vivo* models (23).

In cell-based assays, aqueous extracts of HC inhibited IBV infection by more than 90% in both Vero and chicken embryo kidney (CEK) cells and markedly reduced virus-induced apoptosis (23). Importantly, protective efficacy was also observed *in vivo*. All specific pathogen–free (SPF) chicken embryos treated with HC extract survived IBV challenge without detectable lesions, and in IBV-infected chicks, treatment increased survival rates to over 50%, significantly exceeding those of untreated controls (23). These findings indicate that HC exerts direct antiviral effects *in vitro* while enhancing host resistance and reducing disease severity in vivo.

Beyond the IBV-specific studies described above, HC has been reported to inhibit the RNA-dependent RNA polymerase (RdRp) activity of SARS-CoV (49). This is indirect, cross-coronavirus evidence and should be regarded as a hypothesis for IBV-specific target validation rather than as confirmation that HC inhibits IBV RdRp. Collectively, the available IBV-specific studies support antiviral activity of HC in the tested cell, chicken-embryo, and chick models, whereas the proposed RdRp mechanism remains unconfirmed for IBV.

#### 2.3.2 Garlic (*Allium sativum*)

Garlic (*Allium sativum*), a plant rich in organosulfur compounds, is widely recognized for its antibacterial, antiviral, and immunomodulatory properties and has been evaluated for activity against IBV (24, 50). Using a chicken embryo infection model, Mohajer Shojai et al. (24) demonstrated that garlic extract significantly improved survival outcomes in SPF embryos challenged with IBV. Notably, protective effects were observed whether the extract was co-administered with the virus or applied after infection, indicating antiviral activity across multiple stages of infection.

The study further showed that garlic extract reduced the pathogenicity of two genetically distinct IBV strains, M41 and 4/91, showing activity against both tested strains (24). Although the precise molecular mechanisms underlying these effects remain to be elucidated, non-IBV and general pharmacological studies have proposed that organosulfur constituents such as allicin may inactivate viral particles or interfere with viral replication processes (50, 51). This mechanism-level evidence is indirect with respect to IBV and requires confirmation in IBV-specific models.

### 2.4 Herbs and extracts from other sources

In addition to traditional Chinese medicinal herbs, several plant-derived extracts from non-TCM sources have demonstrated anti-IBV activity in screening studies, underscoring the diversity of natural products with potential applications in IBV prevention and control (44).

#### 2.4.1 Essential oils from *Mentha piperita*, *Thymus vulgaris*, and *Origanum vulgare*

Essential oils and alcoholic extracts derived from *Mentha piperita* (peppermint), *Thymus vulgaris* (thyme), and *Origanum vulgare* (oregano) have exhibited notable anti-IBV activity *in vitro* (11, 44, 52). In a comparative study of 15 medicinal plant extracts, Lelešius et al. reported that extracts from peppermint, thyme, and oregano displayed high selectivity indices (SI > 60), where the selectivity index is generally defined as the ratio of the 50% cytotoxic concentration (CC₅₀) to the 50% effective or inhibitory concentration (EC₅₀/IC₅₀). A high SI therefore indicates a relatively broad in vitro safety window, reflecting antiviral efficacy at concentrations well below those causing cytotoxicity (44).

At concentrations corresponding to 25% of the half-maximal cytotoxic concentration (CC₅₀), peppermint and thyme extracts completely suppressed IBV-induced cytopathic effects and plaque formation, achieving 100% plaque inhibition. Within a concentration range of 0.125–0.25 × CC₅₀, these extracts reduced IBV titers in culture supernatants by 2–5 log₁₀, as determined by TCID₅₀ assays (44). Notably, qRT-PCR analysis revealed residual intracellular viral RNA despite substantial reductions in extracellular viral yield, indicating that while these extracts markedly limit productive infection, they may not fully eliminate intracellular viral genomes.

These observations suggest that certain plant-derived essential oils can reduce IBV replication or release. However, further dose optimization, mechanistic validation, and safety assessment are required before their practical application in poultry production (10, 44).

#### 2.4.2 Hypericin

Hypericin is a major naphthodianthrone constituent of *Hypericum perforatum* (St John’s wort), although the whole extract also contains pseudohypericin, flavonoids, hyperforin, and other bioactive compounds (20, 53). Methods based on ultrasonic extraction, silica-gel purification, HPLC, and UHPLC have been developed for hypericin determination and may support chemical characterization and batch standardization of experimental preparations (54, 55). In IBV-infected chicken embryo kidney (CEK) cells, hypericin exhibits dose-dependent antiviral effects when administered post-infection, accompanied by marked suppression of virus-induced apoptosis and excessive reactive oxygen species (ROS) production (21).

**Figure 4:**
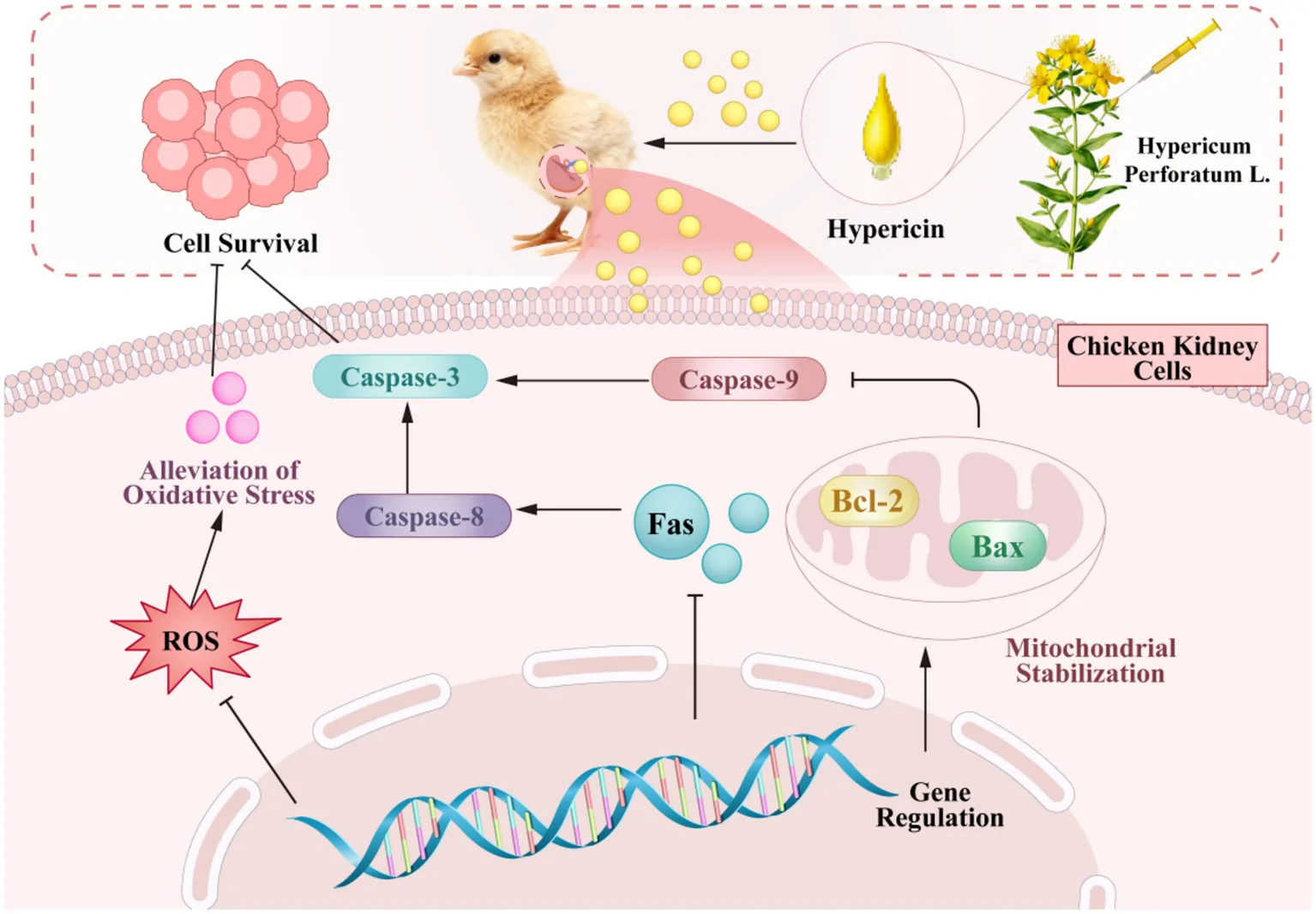
Protective effects of hypericin against IBV-induced apoptosis and oxidative stress. Hypericin protects IBV-infected chicken kidney cells by suppressing Fas- and caspase-dependent apoptotic signaling, modulating the Bax/BCL-2 mitochondrial pathway, and reducing intracellular reactive oxygen species (ROS), thereby enhancing cell survival.

Mechanistically, hypericin downregulates the expression of pro-apoptotic mediators, including Fas, Bax, and Caspase-3/8, while concurrently upregulating the anti-apoptotic protein BCL-2. In parallel, hypericin effectively attenuates IBV-induced oxidative stress by limiting intracellular ROS accumulation (21). Together, these findings suggest that hypericin acts mainly through cytoprotective mechanisms, preserving cell viability by reducing programmed cell death and oxidative damage. Rather than directly targeting viral replication machinery, hypericin may reduce the cellular damage that contributes to disease progression during IBV infection (Figure 4).

#### 2.4.3 Luteolin and myricetin

Luteolin and myricetin are widely distributed flavonoids present in various medicinal plants and dietary sources. Direct IBV-specific evidence is available for myricetin, whereas the evidence for luteolin is indirect (22, 56). *In vitro* studies indicate that myricetin directly targets the active site of the IBV papain-like protease (PLpro), inhibiting its deubiquitinating activity and thereby disrupting viral polyprotein processing required for replication (22). Consistent with this mechanism, myricetin treatment reduces intracellular IBV RNA levels and attenuates virus-induced cytopathic effects.

In contrast, non-IBV evidence suggests that luteolin may exert host-directed anti-inflammatory effects, including modulation of the GSK3β/Nrf2 pathway (56). No direct evaluation of luteolin in IBV-infected cell cultures, chicken embryos, or live chickens was identified in the literature included in this review. Luteolin should therefore be regarded as a candidate for IBV-specific investigation rather than as an established anti-IBV agent.

Recent studies collectively indicate that single medicinal herbs and their bioactive constituents can affect IBV infection through diverse mechanisms. Isolated compounds often have relatively defined molecular targets or pathways. For example, baicalin and phillygenin inhibit IBV replication by inducing stress granule formation, forsythoside A reduces virus-induced apoptosis and interferes with viral entry, hypericin protects infected cells by suppressing oxidative stress and programmed cell death, and myricetin directly inhibits viral replication by targeting PLpro.

By contrast, intact herbs and crude extracts usually contain multiple constituents and may act through broader, less clearly separated mechanisms. Extracts from *Houttuynia cordata* and garlic, for instance, appear to suppress viral replication while also enhancing host resistance or reducing virus-induced cellular damage. Overall, studies of single herbs and their active constituents provide important insight into the material basis and mechanistic diversity of natural anti-IBV agents. They also provide a foundation for the rational design of compound formulations and adjunctive strategies for IBV prevention and control.

## 3 Research on Chinese herbal compound preparations for the prevention and treatment of IBV

Compared with isolated compounds, Chinese herbal compound preparations are designed to combine multiple medicinal components with potentially complementary activities. This feature makes them relevant to complex viral diseases such as infectious bronchitis, where viral replication, inflammatory injury, immune dysregulation, and secondary complications may occur simultaneously. In the context of IBV prevention and control, several classical prescriptions and modern veterinary herbal formulations have shown protective effects in experimental or field-related studies (57).

These formulations often include herbs traditionally used for febrile respiratory disorders, cough, phlegm accumulation, impaired airway function, or qi deficiency. In modern pharmacological terms, they are commonly investigated for antiviral, anti-inflammatory, expectorant, immunomodulatory, and tissue-protective effects. The following sections summarize representative studies on Chinese herbal compound preparations for IBV control.

### 3.1 Maxing Shigan Tang and its modified formulations

Maxing Shigan Tang (MXSG), originally described in the classical medical text Shanghan Lun, consists of Ephedra herb, apricot kernel, gypsum, and licorice root. Traditionally, MXSG has been used to treat febrile respiratory disorders characterized by cough, dyspnea, and lung heat. In modern pharmacological studies, MXSG and related formulations are mainly discussed in relation to anti-inflammatory, antiviral, airway-protective, and immunomodulatory effects (58). Although findings from human respiratory viral infections, including SARS-CoV-2, are indirect with respect to IBV (9), they provided contextual rationale for subsequent IBV-specific investigation of MXSG and its modified formulations.

Liu et al. reported that MXSG has been applied for long-term prevention and treatment of infectious bronchitis in commercial chicken farms in China, with observable clinical benefits (58). However, traditional formulations present practical limitations for large-scale poultry production, including high dosage requirements and challenges in component standardization. To address these issues, the authors employed the traditional Chinese medicine inheritance calculation platform combined with network pharmacology to optimize the original formula, resulting in two modified formulations, designated MXSG-mix1 and MXSG-mix2.

In specific pathogen–free (SPF) chicken challenge models, the modified MXSG formulations exhibited superior anti-IBV efficacy compared with the original prescription. Treatment significantly reduced IBV loads in tracheal and lung tissues while concomitantly increasing immune organ indices and IBV-specific antibody levels, indicating enhanced antiviral and immunomodulatory effects. Network pharmacology analysis further identified 28 putative active compounds within MXSG-mix acting on 47 host targets associated with viral replication, inflammation, and apoptosis. RT-qPCR analysis indicated treatment-associated changes in AKT1, BCL2, and CASP3 mRNA expression. However, because the direction of BCL2 regulation was reported inconsistently in the original study and a causal relationship between these expression changes and apoptosis was not established, these findings should be interpreted as preliminary evidence of apoptosis-associated pathway modulation rather than proof that BCL2 downregulation suppresses IBV-induced apoptosis (58).

Collectively, MXSG and its optimized formulations exert anti-IBV activity through multi-component and multi-target mechanisms, integrating antiviral, immunomodulatory, and cytoprotective effects. These findings support further evaluation of MXSG and its optimized formulations as potential adjunctive interventions within integrated IBV control programs, particularly under conditions where vaccine-induced protection is incomplete or strain mismatched (58).

### 3.2 Ban-Qin-Fei-re-Qing Oral liquid

Ban-Qin-Fei-Re-Qing (BQ) oral liquid is a modern Chinese veterinary herbal formulation developed for respiratory diseases. It is characterized by the combined use of Isatis indigotica (Banlangen) and Scutellaria baicalensis (Huangqin), which are traditionally used to relieve lung heat and inflammation-related respiratory symptoms (59, 60). The formulation contains eight components, including Banlangen, Huangqin, gypsum, Menispermum dauricum, *Platycodon grandiflorus*, *Aster tataricus*, borax, and borneol, and is investigated for lung-protective, expectorant, anti-inflammatory, and immunomodulatory effects (59).

Within the formula, Banlangen serves as the principal antiviral component, exhibiting broad-spectrum activity against respiratory viruses, whereas Huangqin functions as a key adjunct with established antiviral and anti-inflammatory properties (60). Ma et al. systematically evaluated BQ using chicken embryo and *in vivo* chicken infection models (60). BQ treatment significantly reduced mortality and lesion severity in IBV-infected embryos in a dose-dependent manner. In chick trials, oral administration of BQ markedly alleviated clinical symptoms of infectious bronchitis even at low concentrations (2.5 mL/L), indicating favorable therapeutic efficacy.

Mechanistically, transcriptomic analysis showed that BQ treatment modulated host gene expression profiles associated with antiviral immune pathways, particularly the JAK/STAT and type I interferon (IFN) signaling cascades. These findings suggest that the protective effect of BQ is closely associated with enhancement of IFN-mediated innate immune responses (60). Overall, BQ appears to exert protective effects through the combined or complementary actions of multiple components and host-directed immunomodulation. However, the contribution of individual components and the presence of pharmacological interactions or synergy have not been directly established. Further studies are needed to clarify these relationships and evaluate the translational potential of the formulation.

### 3.3 Shegan Dilong powder and combination therapies

Shegan Dilong Powder (SGDL), also known as Gandidilong Powder, is a modern Chinese veterinary formulation used for avian respiratory disorders (61). Its core components include *Belamcanda chinensis* (Shegan), traditionally associated with anti-inflammatory and throat-relieving effects, and Pheretima (Dilong), which is used in Chinese medicine to relieve airway obstruction and dyspnea (62–64). Reported variations in formulation composition likely reflect attempts to optimize clinical performance.

Feng et al. evaluated the preventive and therapeutic efficacy of SGDL alone or in combination with doxycycline in IBV-infected chicks (63). While SGDL monotherapy moderately reduced viral loads and tissue damage, the combined-treatment group showed greater improvements in the reported outcomes. The combination group exhibited higher serum anti-IBV antibody titers and tracheal IgA levels, lower IBV RNA loads in the trachea and lungs, and less severe histopathological lesions than the single-treatment and control groups.

At the immunological level, the combined-treatment group showed reduced expression of the pro-inflammatory cytokines IL-1β, IL-6, and TNF-*α*, together with increased IFN-*γ* expression in respiratory tissues. These changes were accompanied by reduced viral RNA loads and improved histopathological outcomes. However, the cited study did not directly establish the individual contributions of SGDL and doxycycline to the outcomes observed with the combination regimen. Accordingly, the antiviral, immunological, and tissue-protective findings should be presented as effects observed with the combined treatment rather than being attributed mainly to SGDL.

These findings suggest that the SGDL–doxycycline combination regimen may provide adjunctive benefits under the experimental conditions examined. However, they do not establish a general advantage of routinely combining herbal preparations with antimicrobial agents. In accordance with antimicrobial-stewardship principles, doxycycline should be used only when there is an appropriate veterinary indication, such as suspected or confirmed secondary bacterial infection, and should not be routinely recommended for IBV infection alone.

### 3.4 Compound throat antiviral agent

Integrative approaches combining metabolomics and network pharmacology have also been used to guide the development of compound formulations. Using data-mining strategies, Liu et al. developed a Compound Throat Antiviral agent (CTA) composed of multiple traditional Chinese medicine components for the prevention and treatment of IBV infection in chickens (65).

CTA treatment reduced IBV loads in both *in vitro* and *in vivo* models and improved growth performance and immune indices in infected chickens. Metabolomic analyses indicated that CTA modulates host metabolic pathways, while network pharmacology suggested that its activity may involve multi-target antiviral effects. Together, these findings illustrate how omics-based and computational approaches can support the rational development of Chinese veterinary anti-IBV compound preparations.

### 3.5 Combined strategies of herbal active components and nutritional additives

Beyond traditional compound formulations, recent studies have explored combined strategies integrating herbal-derived active components with functional nutritional additives for IBV prevention and control. One representative example is a plant essential oil mixture (PEO) composed of cinnamaldehyde (CA) and glycerol monolaurate (GML) at a defined ratio, which has been evaluated for both prophylactic and therapeutic efficacy against IBV (66).

Administration of PEO, either prior to or following IBV challenge, significantly reduced clinical symptom scores across all dosage groups compared with untreated controls, with the most pronounced effects observed in the medium- and high-dose groups. On the seventh day after administration, the clinical recovery rates–defined as the proportions of diseased chickens showing complete or near-complete disappearance of clinical signs together with a ≥ 95% reduction in symptom score–were 92.31 and 91.67% in the medium- and high-dose groups, respectively (66). In addition, PEO treatment reduced IBV viral mRNA levels in the trachea and kidneys compared with untreated controls and the ribavirin-treated group. In parallel, PEO treatment enhanced immune organ indices (e.g., bursa of Fabricius and spleen) and increased IBV-specific antibody titers. Cytokine profiling further revealed increased IL-4 and IFN-*γ* levels together with reduced IL-6 expression (66). The increase in IL-4 is consistent with a Th2-associated immunoregulatory response that may support humoral immunity. In contrast, IFN-γ is a type II interferon associated primarily with antiviral and cell-mediated immune responses and may also promote inflammation; it should therefore not be classified simply as an anti-inflammatory cytokine. Accordingly, the reduction in IL-6, rather than the increase in IFN-γ, provides more direct evidence of attenuation of excessive inflammatory signaling.

Mechanistically, CA-mediated interference with receptor-mediated viral endocytic entry and GML-mediated virucidal activity against enveloped viruses are supported by constituent-level or non-IBV evidence and were not directly demonstrated in the IBV chicken study. These mechanisms should therefore be regarded as indirect hypotheses for IBV. The IBV-specific study supports protective and immunomodulatory effects of PEO in chickens, but does not directly confirm the proposed CA- and GML-specific mechanisms (66). Accordingly, PEO may provide complementary antiviral and immunomodulatory effects, but its constituent-specific mechanisms require IBV-specific validation.

Owing to their complex composition and broad biological activities, Chinese herbal compound preparations may offer practical advantages as adjunctive strategies for IBV management. These formulations have the potential to reduce viral burden, alleviate clinical symptoms, support tissue recovery, and limit complications such as secondary bacterial infections following IBV challenge. Further investigation into interactions among components, their combined or complementary effects, quality control, pharmacological mechanisms, and optimized formulation strategies will be essential for developing safe and effective herbal interventions for integrated IBV prevention and control (Table 3). Formal claims of synergy will require experimental designs that distinguish individual-component effects from additive or interactive effects.

**Table 3:** Experimental and computational evidence for traditional Chinese medicine formulae relevant to IBV infection.

Formula name	Chinese medicine composition	Experimental model/Cell	Tested IBV strain	Mechanism of action	Reference
SHL	*Lonicera japonica*/honeysuckle flower, Scutellaria baicalensis, and *Forsythia suspensa*	In silico analysis: network pharmacology, machine learning, molecular docking, and molecular dynamics; non-IBV cell-culture evidence against human adenovirus type 3	Not applicable; no IBV experimental model	Predicted candidate constituents and targets, including IL6 and BCL2; computational and hypothesis-generating evidence only	(71, 72)
MXSG	MXSG: Ephedra, bitter almond, gypsum/plaster, and licorice. MXSG-mix1: licorice, bitter almond, Ephedra, Platycodon, and *Fritillaria*. MXSG-mix2: licorice, bitter almond, Ephedra, Platycodon, *Stemona japonica*, and Rhizoma Belamcandae.	SPF chicken embryos and live SPF chickens	Pathogenic IBV-M41 strain/IBV-M41 strain, batch number AV1511	IL-4↑; TNF-α↓; IFN-γ↑; IL-10↑; IL-6↓; SOD↑; T-AOC↑; NO↑	(58)
BQ	*Isatis indigotica* root, Scutellaria baicalensis root, gypsum, *Menispermum dauricum*, *Platycodon grandiflorus*, *Aster tataricus*, borax, and borneol.	SPF chicken embryos; chickens	IBV JS/2010/12 strain	IFN-α↑; IFN-β↑; p-STAT1↑; JAK1↑; TYK2↑	(60)
SGDL + doxycycline combination regimen	*Belamcanda chinensis* (L.) Redouté radix, *Pheretima*, *Glycyrrhiza uralensis* Fisch radix, *Schisandra chinensis*, Menispermi Rhizoma radix, *Platycodon grandiflorus*, and Dark Plum Fruit, SGDL was administered together with doxycycline in the cited combination-treatment group	IBV-infected broiler chickens	IBV-M41 strain	Combination-treatment findings: serum anti-IBV antibody titers↑; tracheal IgA↑; tracheal and lung IBV RNA↓; IL-1β↓; IL-6↓; TNF-α↓; IFN-γ↑. The individual contributions of SGDL and doxycycline were not directly established.	(63)
CTA	*Rehmannia glutinosa*, *Fritillaria* fritillary, and other natural plant components identified through the Traditional Chinese Medicine Inheritance System.	CEK cells; SPF chicken embryos; chickens (challenge test)	IBV-M41 strain	IL-4↑; IFN-γ↑; IL-10↑; TNF-α↓; IL-6↓; IL-1β↓; EGFR↓; STAT3↓; CDK2↓; FoxO↓	(65)
PEO	Plant essential oil mixture composed of cinnamaldehyde (CA) and glycerol monolaurate (GML)	Chickens	IBV-M41 strain	IFN-γ↑; IL-4↑; IL-6↓	(66)

↑ indicates upregulation and ↓ indicates downregulation. Evidence derived solely from computational analyses or non-IBV experimental systems is indirect and is not equivalent to anti-IBV efficacy demonstrated in IBV-infected cells, chicken embryos, or live chickens. For the SGDL-related entry, the virological, immunological, cytokine, and pathological outcomes were observed with the SGDL–doxycycline combination regimen and should not be attributed to SGDL alone.

### 3.6 Interventions supported mainly by indirect, computational, or non-IBV evidence

Several herbal agents and formulations discussed in the literature lack direct experimental validation in IBV-infected cell cultures, chicken embryos, or live chickens. For these interventions, current evidence is derived predominantly from computational analyses, non-IBV experimental models, general pharmacological studies, or extrapolation from compositionally related formulations. They should therefore be regarded as hypothesis-generating candidates rather than established anti-IBV interventions.

For single herbs and isolated constituents, only chlorogenic acid—a defined component of *Lonicera japonica*—has been directly evaluated in IBV-infected CEK cell cultures and live chickens, where it reduced viral RNA levels, ameliorated respiratory tissue injury, and modulated MDA5-, TLR7-, and interferon-associated responses (67). However, evidence obtained for an isolated constituent cannot be transferred directly to the whole herb, because extract composition, bioavailability, effective dose, and pharmacological activity may differ. Whole-herb *Lonicera japonica* preparations therefore remain sources of candidate compounds rather than experimentally confirmed anti-IBV interventions. For matrine and oxymatrine, the available evidence is derived predominantly from general pharmacological studies and non-IBV experimental systems (68–70). No adequately characterized studies evaluating these alkaloids in IBV-infected cell cultures, chicken embryos, or live chickens were identified in the literature reviewed; their relevance to IBV is therefore indirect.

For compound formulations, Shuanghuanglian is supported only by computational analyses (network pharmacology, machine learning, molecular docking, and molecular dynamics simulations) and non-IBV cell-culture testing against human adenovirus type 3 (71, 72). These analyses identified candidate constituents and predicted targets, including IL6 and BCL2, but no validation was performed in IBV-infected models. Jinhua Qinggan Granules have been evaluated solely in a non-IBV acute lung injury model, in which regulation of the TLR4/MyD88/NF-κB pathway was reported (73); no IBV-specific study was identified. Ma Xing Shi Gan Weijing Yin has not been directly tested in IBV-infected models; the existing evidence is extrapolated from a compositionally distinct modified Ma Xing Shi Gan decoction evaluated in IBV-infected chicks (74).

Collectively, these interventions represent candidates for future IBV-specific investigation. Their inclusion here serves to delineate the boundary between experimentally supported interventions and those requiring rigorous validation in IBV-permissive cell cultures, chicken embryos, and live-chicken challenge models.

## 4 Common mechanisms underlying traditional Chinese medicine–mediated protection against IBV

The anti-IBV activity of traditional Chinese medicine (TCM) does not rely on a single molecular target or signaling pathway. Instead, it represents an integrated, multi-layered defense network that operates across distinct stages of viral infection.

From a mechanistic perspective, TCM-mediated protection against IBV can be systematically organized into two broad but interconnected categories: direct virus-targeted effects and host-directed effects (Tables 4, 5). Direct virus-targeted effects act on virions, viral structural components, or viral enzymes. These effects include physical or chemical inactivation of virions, disruption of viral envelope integrity, blockade of viral attachment or entry, and inhibition of viral enzymes such as papain-like protease (PLpro) and RNA-dependent RNA polymerase (RdRp).

**Table 4:** Host-directed regulatory mechanisms involved in TCM-mediated protection against IBV.

Dominant mechanism category	Mechanistic axis	Representative herbal components/formulations	Key molecular targets/pathways	Stage of IBV infection	Functional consequence
Host-directed immunomodulatory effect	Type I interferon signaling	Astragalus polysaccharide (APS); *Radix Isatidis* polysaccharide (RIP); baicalin; Ban-Qin-Fei-Re-Qing oral liquid; CTA	MDA5, TLR3, IRF7 (RIP-associated); IRF3 (baicalin-associated); IFN-β, JAK1, TYK2, STAT1	Early–mid stage	Establishment of antiviral state; suppression of viral replication
Host-directed stress-adaptive antiviral effect	Stress granule-mediated translational arrest	Baicalin; phillygenin	PKR, eIF2α, G3BP1, stress granule assembly	Viral protein synthesis	Inhibition of IBV protein translation through host-cell translational control
Host-directed cell-fate regulatory effect	Context-dependent autophagy- and apoptosis-associated regulation	Forsythoside A; Maxing Shigan Tang and its optimized formulations (MXSG, MXSG-mix)	PI3K, Akt, mTOR, Beclin-1, LC3-II, Caspase-3, BCL-2; AKT1, BCL2, and CASP3 mRNA for MXSG-mix	Viral replication and tissue-injury phase	Modulation of cell-survival, apoptosis-associated, and autophagy-associated responses; complete autophagic flux was not established for forsythoside A, and the direction and functional consequence of BCL2 regulation remain uncertain for MXSG-mix.
Host-directed cytoprotective effect	Oxidative stress regulation	Astragalus polysaccharide (APS); hypericin; *Houttuynia cordata* extract	ROS, Nrf2, antioxidant enzymes, SOD, GSH-Px	Host injury phase	Hypericin reduced ROS in IBV-infected chicken embryo kidney cells. Nrf2- and antioxidant-enzyme-related effects attributed to APS and *H. cordata* remain indirect and require IBV-specific validation
Host-directed anti-inflammatory effect	Inflammatory signaling regulation	Astragalus polysaccharide (APS); *Radix Isatidis* polysaccharide (RIP); forsythoside A; SGDL + doxycycline combination regimen	NF-κB, IL-1β, IL-6, IL-8, TNF-α. For the SGDL–doxycycline regimen, the cited study directly reported cytokine-expression changes but did not directly establish NF-κB pathway modulation.	Mid–late infection/tissue injury phase	Reduction of excessive cytokine production and inflammatory tissue damage. For the SGDL-related evidence, reduced IL-1β, IL-6, and TNF-α expression was observed in the combination-treatment group and should not be attributed to SGDL alone.

For MXSG-mix, changes in AKT1, BCL2, and CASP3 mRNA expression were reported, but the direction of BCL2 regulation was internally inconsistent in the original study. These findings therefore indicate preliminary pathway involvement rather than a confirmed BCL-2-mediated anti-apoptotic mechanism. The SGDL-related anti-inflammatory evidence summarized in this table refers to the SGDL–doxycycline combination regimen. The individual contribution of SGDL was not directly established.

**Table 5:** Direct virus-targeted and dual/multi-target mechanisms involved in herbal anti-IBV activity.

Dominant mechanism category	Mechanistic axis	Representative herbal components/formulations	Key molecular targets/pathways	Stage of IBV infection	Functional consequence
Direct virus-targeted effect	Direct virucidal activity	Elderberry extract; garlic extract; glycerol monolaurate-containing PEO	Viral envelope; viral structural integrity; enveloped-virus inactivation	Pre-entry	Reduction of IBV infectivity before cellular attachment has been demonstrated for elderberry. The corresponding garlic- and GML-related virucidal mechanisms remain candidate hypotheses requiring IBV-specific validation
	Viral attachment and entry blockade	Forsythoside A; cinnamaldehyde-containing PEO; *Houttuynia cordata* extract; sulfated polysaccharides with coronavirus-relevant evidence	IBV S1 subunit; membrane fusion processes; receptor-mediated endocytic entry; viral attachment factors	Attachment/entry	IBV-specific entry inhibition has been demonstrated for forsythoside A and reported for *H. cordata*. Cinnamaldehyde- and sulfated-polysaccharide-related entry mechanisms remain candidate hypotheses for IBV
	Viral enzyme or replication machinery inhibition	Myricetin; *Houttuynia cordata* extract	IBV PLpro; coronavirus RdRp-related replication	Intracellular replication	Inhibition of IBV PLpro by myricetin is supported by IBV-specific evidence. The proposed *H. cordata*–RdRp mechanism remains an indirect candidate mechanism for IBV
Dual or multi-target effect	Combined direct antiviral and host-directed regulation	Baicalin; forsythoside A; *Houttuynia cordata* extract; CTA; PEO	Viral entry/replication targets plus IFN signaling, NF-κB, oxidative stress, apoptosis, or cytokine pathways	Multiple stages	Reductions in viral burden and host injury have been reported in IBV-specific models, but individual molecular-mechanism assignments should be interpreted according to the stated evidence level

By contrast, PKR/eIF2α/G3BP1-dependent stress-granule formation is a host-cell response. Although activation of this pathway suppresses viral protein synthesis, it acts through host translational control and is therefore classified as a host-directed stress-adaptive antiviral mechanism rather than a direct effect on virions or viral proteins. Other host-directed effects include enhancement of innate antiviral immunity, including pattern-recognition receptor activation, type I interferon signaling, and JAK–STAT pathways; augmentation of adaptive immune responses; regulation of inflammatory signaling; restoration of redox homeostasis; and modulation of autophagy- and apoptosis-associated responses.

Importantly, direct virus-targeted and host-directed effects are not mutually exclusive, and an individual compound may exhibit both types of activity. For example, baicalin can directly inactivate IBV particles, representing a direct virus-targeted effect, while its activation of the PKR/eIF2α/G3BP1 stress-granule pathway and type I interferon signaling represents host-directed activity. Similarly, forsythoside A directly binds to the IBV S1 subunit to interfere with viral entry while also modulating host PI3K/Akt/NF-κB-associated responses. Thus, the stage of the viral life cycle affected by an intervention should be distinguished from whether the underlying mechanism acts directly on the virus or indirectly through the host cell.

### 4.1 Direct virus-targeted antiviral effects

Certain TCM-derived components exert direct antiviral activity by physically or chemically inactivating IBV particles before host cell entry. Such virus-targeted actions primarily involve disruption of viral structural integrity, leading to loss of infectivity and effective blockade of early infection events.

For instance, polyphenolic compounds in elderberry (*Sambucus nigra*) extracts have been shown to damage the IBV envelope, leading to loss of viral infectivity before cellular attachment (19). Similarly, sulfur-containing compounds in garlic (*Allium sativum*) have been proposed, on the basis of non-IBV and general antiviral evidence, to modify viral envelope- or capsid-associated proteins. This is an indirect candidate mechanism that has not been demonstrated for IBV; the direct IBV-specific evidence for garlic is limited to the protective effect observed in the chicken-embryo model (24). At the formulation level, Ban-Qin-Fei-Re-Qing (BQ) oral liquid reduced IBV titers and attenuated tissue lesions in chicken embryo models in a dose-dependent manner (60). However, this effect should be interpreted cautiously, because reduced viral titers in embryos may reflect combined direct antiviral and host-mediated protective effects rather than purely virucidal activity.

Collectively, these findings indicate that some herbal components can reduce IBV infectivity at the extracellular or early-entry stage. This mode of action may lower the initial viral burden and complement downstream host-directed antiviral responses.

### 4.2 Interference with viral entry and replication

Several herbal-derived compounds have been reported to interfere with different stages of the IBV life cycle, including viral attachment, entry, genome replication, and host processes that support viral propagation. Rather than acting on a single viral factor, these compounds may restrict productive infection at both early and intracellular stages.

#### 4.2.1 Blocking viral attachment and entry

Viral attachment and entry constitute the first obligate step in IBV infection. IBV initiates infection primarily through interaction of its spike (S) protein with *α*-2,3-linked sialic acid receptors on host cell surfaces (75). Several TCM-derived components effectively interfere with this process, thereby preventing viral internalization.

Forsythoside A directly binds to the S1 subunit of the IBV S protein, competitively blocking virus–receptor interactions and preventing viral entry (42). In parallel, plant-derived essential oil components such as cinnamaldehyde (CA) inhibit receptor-mediated endocytic entry, potentially by altering membrane fluidity and endosomal acidification (76). This remains an indirect hypothesis for IBV and has not been confirmed in an IBV-specific entry assay. Extracts of *Houttuynia cordata* (HC) have likewise been reported to suppress early attachment and entry events, resulting in reduced downstream viral replication (23). Together, these agents may reduce the efficiency of IBV attachment or entry, thereby limiting access to susceptible host cells.

#### 4.2.2 Disruption of viral genome replication and protein expression

Beyond entry blockade, some herbal-derived compounds may interfere with viral replication machinery. Myricetin has been reported to target the IBV papain-like protease (PLpro), inhibiting its deubiquitinating activity and impairing viral polyprotein processing required for replication (22). In addition, *Houttuynia cordata* extracts have been shown to inhibit the RNA-dependent RNA polymerase (RdRp) activity of SARS-CoV, providing indirect evidence for potential activity against conserved coronavirus replication processes (49).

Collectively, these findings suggest that selected herbal components may constrain viral replication by targeting viral enzymatic functions. However, cross-coronavirus evidence should be further validated in IBV-specific models.

### 4.3 Induction of antiviral innate and adaptive immunity

An important feature of TCM-mediated protection against IBV is its potential to influence both innate and adaptive immune responses. Rather than indiscriminately amplifying immunity, TCM-derived components have been associated with enhanced pathogen sensing, interferon-related signaling, stress-adaptive translational suppression, and vaccine-associated humoral and cellular immune responses. However, the available studies do not directly establish the induction or durability of immunological memory. Together, these immune-related effects represent an important component of the proposed host-directed protective framework.

#### 4.3.1 Enhancement of innate antiviral signaling

Polysaccharides derived from herbal medicines represent potent activators of innate antiviral immunity (77). Isatis indigotica polysaccharide (RIP) markedly upregulates intracellular pattern-recognition receptors (PRRs), including MDA5 and TLR3, leading to activation of IRF7 and robust induction of type I interferon (IFN-*β*) production (15). This PRR–IRF–IFN axis establishes an antiviral state that restricts early IBV replication.

Compared with single-component interventions, compound formulations may affect a broader range of innate immune pathways. Transcriptomic analyses showed that Ban-Qin-Fei-Re-Qing (BQ) oral liquid was associated with changes in the JAK–STAT and type I IFN signaling pathways, consistent with enhanced interferon-related innate immune responses (60). The duration of these responses was not directly established. Together, these findings suggest that TCM polysaccharides may prime innate antiviral responses by activating pattern-recognition receptor and interferon-related signaling.

#### 4.3.2 Host-directed stress-granule–mediated translational suppression

As a critical downstream effector arm of the type I interferon response, host cells also deploy a more generalized translational control mechanism to restrict viral protein synthesis: the formation of stress granules. Stress granules (SGs) are dynamic, membraneless cytoplasmic structures assembled under various cellular stresses, including viral infection. They sequester mRNAs and translation-associated proteins, thereby suppressing global or selective translation, including that of viral proteins (78, 79). Several flavonoid-related compounds, notably baicalin and phillygenin, activate the host PKR/eIF2α pathway and promote G3BP1-dependent SG assembly in IBV-infected cells, resulting in reduced viral N-protein expression and inhibition of viral replication (16, 18).

Knockdown of G3BP1 markedly attenuates the antiviral activity of baicalin and phillygenin, supporting the functional importance of the PKR/eIF2α/G3BP1 pathway in this response (16, 18). Because PKR, eIF2α, G3BP1, and SG assembly are components of the host-cell stress and translational-control machinery, this mechanism is classified as a host-directed stress-adaptive antiviral effect. Its downstream inhibition of viral protein synthesis does not make it a direct effect on virions or viral proteins.

Notably, the PKR/eIF2α axis is inducible by type I interferon signaling, and baicalin has been reported to affect both the IRF3/IFN-*β* and PKR/eIF2α pathways (16, 17). These findings support a functional connection and possible mechanistic convergence between IFN-related antiviral signaling and SG-mediated translational suppression; however, a reciprocal positive-feedback circuit has not been directly demonstrated. Baicalin therefore exemplifies how a single compound may engage two distinct but functionally connected innate antiviral mechanisms.

#### 4.3.3 Enhancement of adaptive immune responses

Beyond innate immunity, TCM-derived components may also enhance measurable adaptive immune responses. Astragalus polysaccharide (APS), when used as a vaccine adjuvant, significantly increased antigen-specific antibody production, promoted T-lymphocyte proliferation, and increased the expression or secretion of immunoregulatory cytokines, including IL-2 and IFN-*γ* (30). These findings indicate enhanced vaccine-associated humoral and cellular immune responses against IBV; however, the study did not directly evaluate long-term memory B cells, memory T cells, or durable recall responses.

Notably, compound formulations may affect both mucosal and systemic immune responses. For example, chickens receiving the combination of Shegan Dilong Powder and doxycycline showed higher serum anti-IBV antibody titers, increased secretory IgA (sIgA) levels in the tracheal mucosa, and increased IFN-γ expression in lung tissues (63). These findings indicate that enhanced systemic and respiratory mucosal immune responses were observed with the combination regimen; however, the separate contribution of SGDL or doxycycline to these responses was not established.

### 4.4 Regulation of inflammatory responses

IBV infection frequently provokes excessive inflammatory responses characterized by cytokine overproduction, which contributes to tracheal mucosal injury, ciliary dysfunction, and disease progression. This dysregulated inflammation, often described as a cytokine storm, represents a key pathological driver of IBV-associated tissue damage (80).

Anti-inflammatory effects of TCM are mediated not only through direct suppression of pro-inflammatory mediators but also through precise modulation of core inflammatory signaling networks (81). Among these, the NF-κB pathway serves as a central regulatory hub integrating viral sensing, immune activation, and cytokine production. Multiple TCM-derived components exert anti-inflammatory activity by targeting this pathway. For example, Isatis indigotica polysaccharide inhibits NF-κB activation and nuclear translocation, thereby suppressing transcription of downstream inflammatory cytokines (15). Similarly, forsythoside A attenuates inflammatory responses by modulating the upstream PI3K/Akt/NF-κB signaling axis (25).

At the formulation level, compound preparations may exert broader anti-inflammatory effects through the combined regulation of multiple targets. Astragalus polysaccharide (APS) significantly reduces the expression of key pro-inflammatory cytokines, including IL-1β, IL-6, IL-8, and TNF-*α*, in IBV-infected cells (14). Likewise, combined treatment with Shegan Dilong Powder and doxycycline was associated with reduced IL-1β, IL-6, and TNF-α expression in the respiratory tract of infected chickens (63). These cytokine changes should be interpreted as findings from the combination-treatment group rather than as effects established for SGDL alone.

Collectively, these findings indicate that TCM alleviates IBV-induced inflammatory injury through coordinated suppression of excessive cytokine production and fine-tuning of inflammatory signaling pathways. This dual-mode regulation mitigates immunopathology while preserving antiviral defense, thereby providing a critical mechanistic basis for improving disease outcomes during IBV infection.

### 4.5 Regulation of oxidative stress

Viral infection–induced inflammation is frequently accompanied by excessive generation of reactive oxygen species (ROS). Sustained oxidative stress disrupts epithelial integrity, impairs mitochondrial function, and compromises immune cell antiviral capacity, thereby accelerating disease progression (82, 83). In IBV infection, oxidative stress not only contributes directly to tissue injury but also amplifies inflammatory signaling, forming a self-reinforcing pathogenic loop.

Multiple TCM-derived components have been proposed to regulate oxidative stress, but the level of evidence differs among interventions. Hypericin directly reduced intracellular ROS accumulation in IBV-infected chicken embryo kidney cells (21). By contrast, Nrf2 activation attributed to Astragalus polysaccharide and antioxidant effects proposed for Isatis indigotica (Banlangen) and *Isatis tinctoria* (Daqingye), including regulation of superoxide dismutase and glutathione peroxidase, are supported mainly by non-IBV or general pharmacological studies (84–86). These mechanisms should therefore be considered indirect and hypothesis-generating for IBV. If confirmed in IBV-specific models, such antioxidant effects could complement anti-inflammatory and antiviral mechanisms by limiting virus-associated oxidative injury.

### 4.6 Modulation of apoptosis, autophagy, and cell fate

IBV infection triggers both apoptosis and autophagy, influencing host cell fate and tissue integrity. Herbal components can modulate these interconnected processes, thereby limiting virus-induced cellular damage.

Apoptosis is a prominent host cell response to IBV infection and a major contributor to virus-induced tissue injury. Excessive or dysregulated apoptosis exacerbates tissue injury during IBV infection (87, 88). *Houttuynia cordata* extracts reduced apoptosis in IBV-infected cells by over 90% (23). Hypericin suppresses apoptosis by downregulating Fas, Bax, and Caspase-3/8 while upregulating BCL-2 (21). MXSG-mix treatment was associated with changes in AKT1, BCL2, and CASP3 mRNA expression; however, the direction of BCL2 regulation was inconsistent in the original study, and a causal link to apoptosis was not established. These findings therefore indicate preliminary pathway involvement rather than confirmed anti-apoptotic efficacy (58).

Beyond apoptosis, autophagy constitutes another critical cell fate pathway modulated during IBV infection. Autophagy is a multistep degradation process; static changes in LC3-II, Beclin-1, or autophagosome abundance cannot establish complete autophagic flux (89). The functional role of autophagy during IBV infection is context-dependent, varying with cell type, viral strain, and infection stage, and should not be assumed to be uniformly proviral or antiviral (25, 26, 89). In IBV-infected BHK cells, forsythoside A reduced cytopathic effects and was accompanied by changes in PI3K/Akt/NF-κB signaling and in Beclin-1, LC3-II, Caspase-3, and Bax/BCL-2-associated markers (25). These observations indicate modulation of autophagy- and apoptosis-associated responses; however, without direct assessment of autophagic flux, it remains unclear whether the full autophagic process was enhanced, restored, inhibited, or blocked. These findings should not be generalized to other cell types, IBV strains, or infection stages.

Through these coordinated effects, TCM-derived interventions may preserve tissue integrity and mitigate virus-induced cellular damage.

### 4.7 Integrated mechanistic model of herbal protection against IBV infection

Taken together, the protective effects of herbal medicines against IBV can be understood as a coordinated host–virus regulatory network rather than a collection of isolated pathways. This integrated model is organized into three interconnected layers: virus-targeted restriction, antiviral-state reinforcement, and tissue-protective homeostatic regulation.

At the early stage of infection, direct virucidal components and entry-blocking compounds reduce viral attachment, envelope integrity, or spike-mediated internalization, thereby lowering the initial infectious burden. During intracellular replication, both direct virus-targeted and host-directed mechanisms may restrict viral propagation. Some compounds directly inhibit viral enzymes, whereas others activate host-cell responses, including PKR–eIF2α–G3BP1-mediated stress-granule formation, which suppresses viral protein synthesis through host translational control. Current evidence does not establish that autophagy uniformly supports or restricts IBV replication; its functional contribution should therefore be determined separately in each experimental model by assessing complete autophagic flux together with viral replication and cell-injury outcomes.

In parallel, many polysaccharides and compound formulations reinforce host antiviral immunity by activating PRR–IRF–IFN and JAK–STAT signaling, promoting IFN-*β* production, STAT1 activation, and antiviral gene expression. However, IBV-associated pathology is not driven by viral replication alone, but also by excessive inflammation, oxidative stress, and dysregulated cell death. Therefore, herbal interventions also repeatedly affect tissue-protective pathways, including NF-κB-mediated inflammatory regulation, Nrf2-associated antioxidant defense, PI3K/Akt/mTOR-related autophagy control, and Bax/BCL-2/Caspase-dependent apoptosis regulation. Nrf2 activation is included as an indirect candidate mechanism because its current support in this context is derived mainly from non-IBV studies. These pathways collectively reduce immunopathology, preserve epithelial and immune-organ integrity, and improve host resilience during IBV infection.

**Figure 5:**
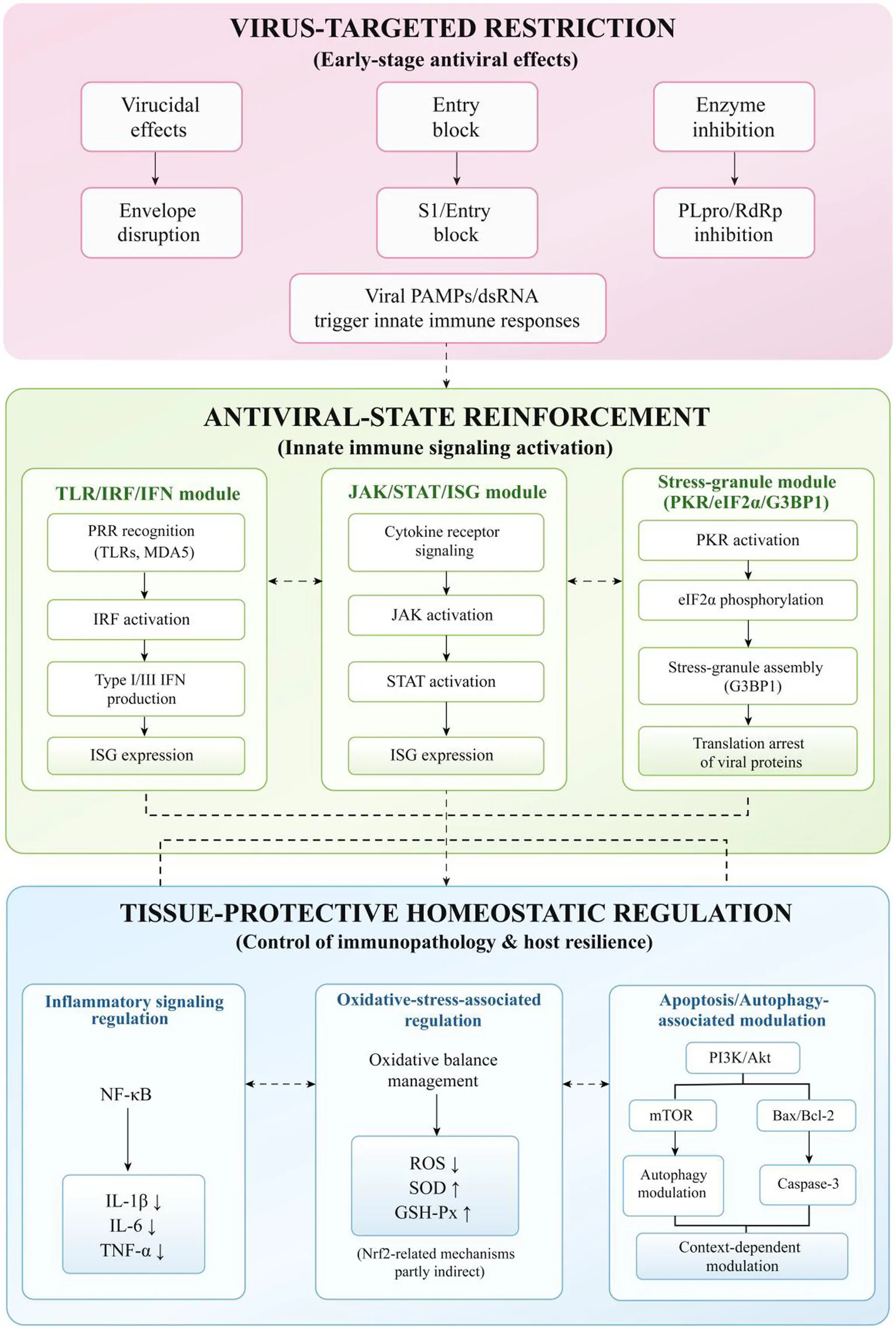
Integrated mechanistic model of herbal protection against IBV infection. The multi-target actions of herbal medicines are illustrated across three interconnected functional layers. Upper panel – Virus-targeted restriction: Representative antiviral strategies include virucidal effects that disrupt viral envelope integrity, entry-blocking compounds that interfere with viral attachment or entry, inhibitors of viral enzymes or replication-related processes. Middle panel – Antiviral-state-related responses: Activation of TLR/IRF/IFN and JAK/STAT modules is associated with type I interferon production and interferon-stimulated gene (ISG) expression, and induction of host-cell translational arrest through the PKR/eIF2α/G3BP1 stress-granule pathway. Lower panel – Tissue-protective and homeostatic regulation: Herbal interventions are associated with modulation of inflammatory responses, oxidative-stress-related pathways, and autophagy- and apoptosis-associated processes, including NF-κB-related cytokine signaling, Nrf2/ROS balance, PI3K/Akt/mTOR-related responses, and Bax/BCL-2/Caspase-3-associated pathways. Connections among these modules indicate possible cross-talk, overlapping regulation, or mechanistic convergence rather than established causal activation, reciprocal feedback circuitry, or pharmacological synergy. Dashed connectors denote potential interconnection or cross-regulation; solid arrows indicate reported directional relationships within specific modules; inhibitory lines indicate suppression. Note: Solid arrows (→) and T-bars (⊣) indicate directional activation and inhibition, respectively, only where these relationships are supported within the cited experimental systems. Dashed connectors indicate potential cross-talk, pathway overlap, or possible cross-regulation and do not imply an established causal direction, reciprocal feedback circuitry, or pharmacological synergy. Dotted arrows indicate putative upstream triggers or conceptual links. See Tables 4–6 for representative compounds, shared molecular targets, and the corresponding levels of evidence. ↑ and ↓ indicate reported increases and decreases, respectively.

Importantly, these signaling hubs do not operate in isolation but are functionally interconnected through extensive cross-talk, forming a coordinated regulatory network rather than a set of parallel pathways (Figure 5). Several cross-connections are particularly relevant to IBV pathogenesis and herbal intervention. First, the NF-κB and Nrf2 pathways are reciprocally regulated through redox-sensitive mechanisms: redox-sensitive cross-talk between NF-κB and Nrf2 may connect inflammatory and antioxidant responses (84). In the IBV literature reviewed here, hypericin directly reduced ROS accumulation (21), whereas APS–Nrf2 involvement remains indirect and requires IBV-specific validation (84). Second, the PI3K/Akt pathway serves as a pivotal junction connecting inflammatory signaling, autophagy, and apoptosis (25, 90). In the forsythoside A study, treatment was accompanied by changes in PI3K/Akt/NF-κB signaling and in Beclin-1, LC3-II, Caspase-3, and Bax/BCL-2-associated markers (25). These observations support coordinated pathway association but do not establish the direction of complete autophagic flux or prove that inflammation, autophagy, and apoptosis were controlled simultaneously through a single causal mechanism. Third, the PKR/eIF2α axis, which is involved in stress-granule formation and translational arrest, can be induced by type I interferon signaling (16, 17). The available findings indicate a functional connection or possible mechanistic convergence between IFN-related signaling and the PKR/eIF2α/G3BP1 stress-granule pathway, but they do not establish reciprocal feedback circuitry. Baicalin, which affects both the IRF3/IFN-*β* and PKR/eIF2α pathways, exemplifies how a single compound may engage two distinct but functionally connected antiviral mechanisms.

Beyond pathway-level overlap, individual herbal compounds and formulae also repeatedly affect shared molecular nodes, indicating mechanistic overlap, partial redundancy, and possible convergence across chemically diverse interventions, as summarized in Table 6 and illustrated in Figure 5. These overlapping targets and pathways should not be interpreted as evidence of pharmacological synergy, which would require direct comparison of individual and combined interventions using an appropriate interaction analysis. The PKR/eIF2*α*/G3BP1 stress granule axis is targeted by both baicalin and phillygenin, two structurally distinct flavonoid-related compounds from different source plants (16, 18). The TLR/NF-κB/IFN-*β* innate immune axis is modulated by both APS and RIP, two polysaccharides derived from different medicinal sources (14, 15). The PI3K/Akt/mTOR/autophagy axis is regulated by forsythoside A, while Bax/BCL-2/Caspase-3-associated apoptotic signaling is modulated by hypericin and forsythoside A (21, 25). MXSG-related formulations have also been associated with changes in AKT1, BCL2, and CASP3 mRNA expression, although the direction and functional consequence of BCL2 regulation remain uncertain (58). Suppression of pro-inflammatory cytokines, including IL-1β, IL-6, and TNF-α, is also repeatedly reported for APS, RIP, the SGDL–doxycycline combination regimen, and several compound formulations (14, 15, 63). For the cited SGDL-related study, these cytokine changes were observed with the combination treatment and were not established as effects of SGDL alone. This pattern of overlapping target engagement suggests that the anti-IBV activity of herbal medicines may not depend only on unique compound-specific mechanisms, but may also reflect their ability to modulate a limited set of host regulatory nodes that are critical for both viral replication and IBV-associated pathology.

**Table 6:** Overlapping molecular targets and shared signaling hubs among representative herbal anti-IBV interventions.

Shared signaling hub/target	Involved compounds/formulae	Source plants (Genus/species)	Chemical class	Functional outcome	Reference
PKR/eIF2α/G3BP1 (Stress granule)	Baicalin, Phillygenin	Scutellaria baicalensis, *Forsythia suspensa*	Flavonoid glycoside, Lignan	Host-cell stress-granule assembly and translational control, resulting in reduced IBV protein synthesis	(16, 18)
TLR/NF-κB/IFN-β (Innate immunity)	APS, RIP	*Astragalus mongholicus*, *Isatis indigotica*	Polysaccharide	IFN-β induction, antiviral state	(14, 15)
JAK/STAT (IFN signaling)	Baicalin, BQ oral liquid	*S. baicalensis*, Multi-herb formula	Flavonoid glycoside, Compound formula	STAT1 activation, ISG expression	(17, 60)
NF-κB (Inflammation)	APS, RIP, Forsythoside A, SGDL + doxycycline combination regimen	*A. mongholicus*, I. indigotica, *F. suspensa*, multi-herb SGDL formula; doxycycline is a non-herbal antimicrobial component	Polysaccharide, Phenylethanoid glycoside, compound herbal formula plus tetracycline-class antibiotic	IL-1β↓; IL-6↓; TNF-α↓. For the SGDL-related study, these cytokine changes were observed with the SGDL–doxycycline combination regimen; the individual contribution of SGDL was not established. Direct regulation of NF-κB by this combination was not demonstrated in the cited study.	(14, 15, 25, 63)
Nrf2/ROS (Oxidative stress)	Hypericin; APS, with the APS–Nrf2 association based on indirect non-IBV evidence	*A. mongholicus*, *Hypericum perforatum*	Polysaccharide, Naphthodianthrone	Hypericin directly reduced ROS in IBV-infected CEK cells. Nrf2 activation attributed to APS is indirect and hypothesis-generating with respect to IBV	(21, 84)
PI3K/Akt/mTOR (Autophagy)	Forsythoside A	*Forsythia suspensa*	Phenylethanoid glycoside	Changes in autophagy-associated markers, including LC3-II↓ and Beclin-1↓; complete autophagic flux was not established, and the functional consequence may be model-dependent	(25)
Bax/BCL-2/Caspase-associated apoptosis	Hypericin, Forsythoside A, MXSG-mix	*H. perforatum*, *F. suspensa*, Multi-herb formula	Naphthodianthrone, Phenylethanoid glycoside, Compound formula	Hypericin and forsythoside A: Caspase-3↓; BCL-2↑; and Bax↓. MXSG-mix: treatment-associated changes in AKT1, BCL2, and CASP3 mRNA expression, with the direction and functional consequence of BCL2 regulation remaining uncertain	(21, 25, 58)

↑ indicates upregulation and ↓ indicates downregulation. For MXSG-mix, the original study reported inconsistent directions of BCL2 regulation across its results and mechanistic interpretation; therefore, no directional BCL-2 effect is assigned in this table. Changes in LC3-II, Beclin-1, or autophagosome abundance should not be interpreted independently as evidence that complete autophagic flux is activated or inhibited. Autophagy-associated effects are considered context-dependent unless validated using flux-sensitive assays and functional pathway perturbation. Stress-granule-mediated translational suppression is classified as a host-directed response because PKR, eIF2α, and G3BP1 are components of the host-cell stress and translational-control machinery. For reference 63, reductions in IL-1β, IL-6, and TNF-α were reported in the SGDL–doxycycline combination-treatment group. The study did not directly establish the contribution of SGDL alone or demonstrate direct modulation of the NF-κB pathway by the combination regimen. The occurrence of shared targets or overlapping pathways across different interventions indicates possible mechanistic convergence but does not by itself demonstrate pharmacological synergy.

From this integrative perspective, the shared host-directed mechanisms of herbal medicines against IBV can be summarized as a multi-target regulatory network. This network operates through coordinated regulation of inflammatory signaling (e.g., NF-κB/cytokines), oxidative stress (e.g., Nrf2/ROS/antioxidants), and apoptosis/autophagy (e.g., PI3K/Akt/mTOR and Bax/BCL-2/Caspase-3), while engaging specialized host stress-adaptive responses, such as PKR/eIF2α/G3BP1-dependent stress granule formation that suppresses viral protein synthesis. Single herbs and active compounds–including APS, RIP, baicalin, phillygenin, forsythoside A, hypericin, myricetin, elderberry, *Houttuynia cordata*, and garlic–engage these shared hubs to produce integrated protective outcomes, including reduced viral entry and replication, reinforced antiviral state, restrained inflammation and oxidative injury, and preserved tissue integrity with enhanced host resilience. This unified model explains how structurally and botanically diverse herbal interventions may produce overlapping antiviral and host-protective effects through multi-target regulation, and provides a conceptual framework for the rational design of multi-herb combinations that maximize mechanistic complementarity while minimizing redundancy.

## 5 Commonly used experimental approaches for evaluating TCM against IBV

Studies investigating the anti-IBV activity of traditional Chinese medicine (TCM) generally adopt multi-level experimental strategies integrating *in vitro* and *in vivo* models with virological and immunological assays. This combined approach enables systematic evaluation of antiviral efficacy while providing mechanistic insights into both virus-targeted and host-directed effects.

### 5.1 *In vitro* cell culture models and antiviral assessment

*In vitro* cell infection models constitute the primary platform for preliminary screening of anti-IBV activity. Commonly used systems include primary chicken embryo kidney (CEK) cells and, for cell-adapted strains such as Beaudette, Vero cells (16, 23, 91). In these models, test compounds are administered prior to or following viral challenge to assess direct antiviral effects.

Viral replication is typically quantified using standard infectivity assays, including plaque-forming unit (PFU) and 50% tissue culture infectious dose (TCID₅₀) assays (92, 93). Reductions in plaque number or TCID₅₀ values are widely accepted indicators of antiviral efficacy, as demonstrated in studies evaluating herbal extracts and bioactive compounds such as hypericin (19, 21). In parallel, cytopathic effect (CPE) observation provides a rapid qualitative measure of antiviral activity, with attenuation of IBV-induced cell fusion, rounding, or detachment indicating effective viral suppression (16, 19, 44).

To distinguish antiviral activity from nonspecific cytotoxicity, cell viability is routinely assessed using assays such as MTT, CCK-8, or trypan blue exclusion. The selectivity index (SI), calculated as the ratio of cytotoxic to antiviral concentrations, is commonly used to estimate the preliminary safety window of candidate compounds (94, 95).

Although *in vitro* models are efficient and reproducible, they cannot fully capture the complexity of host immunity, tissue tropism, pharmacokinetics, or disease progression in chickens. Therefore, these assays are usually complemented by *in vivo* studies to improve biological relevance and support translational interpretation (96).

### 5.2 Chicken embryo and live bird models for *in vivo* evaluation

Because IBV is an avian coronavirus, specific-pathogen-free (SPF) chicken embryos and chicks provide important in vivo models for evaluating traditional Chinese veterinary medicines (97). In the chicken embryo model, 10-day-old SPF embryos are commonly inoculated via the allantoic cavity, which can lead to embryonic death, growth retardation, or characteristic lesions, depending on the viral strain and inoculation dose (98). Test compounds may be administered before infection or at defined time points after challenge. Antiviral activity is then assessed using parameters such as embryo survival rate, lesion score, and embryo index (24, 60). Using this model, Mohajer Shojai et al. (24) demonstrated that garlic extract significantly increased the embryo index in IBV-infected embryos, indicating a protective effect.

Live chicken challenge trials more closely reflect field conditions. SPF chicks (1–2 weeks old) are typically infected via the nasal or tracheal route, followed by drug administration through drinking water or oral gavage. Disease progression is evaluated based on clinical signs, mortality, and gross lesions, while immune responses are assessed by measuring serum anti-IBV antibodies (ELISA), tracheal mucosal IgA, and immune organ indices (bursa of Fabricius, spleen, thymus). Feng et al. (63) reported that chicks receiving combined treatment with Shegan Dilong Powder and doxycycline showed higher systemic anti-IBV antibody and local tracheal IgA responses. These immune outcomes were observed with the combination regimen and were not assigned to either component individually.

Histopathological analysis is commonly incorporated into live bird studies. Tracheal and lung tissues are examined using hematoxylin and eosin (H&E) staining to evaluate inflammation, epithelial damage, and other pathological changes. Collectively, chicken embryo and live bird models provide an integrated assessment of antiviral efficacy, toxicity, immunomodulation, and production performance (e.g., weight gain, feed conversion ratio), and thus remain indispensable tools in the development of traditional Chinese veterinary medicines.

### 5.3 Molecular biology and immunological detection methods

To elucidate the mechanisms underlying the anti-IBV effects of traditional Chinese medicines, a variety of molecular and immunological assays are employed. Quantitative real-time PCR (qRT-PCR) is a core technique for quantifying viral replication and host gene expression (99–101). For IBV detection in infected chickens, Callison et al. (102) developed and evaluated a real-time TaqMan RT-PCR assay targeting the 5′ untranslated region of the IBV genome, providing an IBV-specific molecular method for viral RNA detection. In anti-IBV studies, RT-qPCR assays targeting conserved viral regions, such as the 5′ UTR or N gene, are commonly used to determine viral RNA copy numbers in cells or tissues, enabling precise evaluation of viral inhibition (103). In parallel, qRT-PCR is applied to measure cytokine mRNA levels (e.g., IL-6, IFN-*γ*) as molecular indicators of host immune responses (15, 30, 63).

At the protein level, Western blotting is commonly used to assess IBV structural proteins, such as the N protein. ELISA is used for antibody detection and cytokine quantification, providing evidence for humoral immune responses and inflammatory regulation (104). Immunofluorescence assays (IFAs) can visualize intracellular viral antigens (16, 19), whereas immunohistochemistry (IHC) enables localization of viral antigens or immune-cell markers within tissues (105).

In addition, flow cytometry is widely used to analyze apoptosis, cell cycle distribution, and immune cell subsets. Chen et al. (21) reported that hypericin treatment reduced apoptosis in IBV-infected cells based on Annexin V/PI staining. Flow cytometric analysis of T-cell subsets further facilitates investigation of immunomodulatory effects induced by traditional Chinese medicines.

### 5.4 Omics technologies and computational analysis

With recent technological advances, omics approaches have increasingly been applied to studies of traditional Chinese medicine (TCM) interventions against IBV. Transcriptomics, based on gene microarrays or RNA-seq, is widely used to characterize global host gene expression changes following treatment (106). As noted above, transcriptomic analysis of BQ revealed significant activation of interferon-related signaling pathways (60).

Metabolomics is employed to profile infection- and treatment-associated changes in endogenous metabolites, providing insight into metabolic regulation and symptom alleviation mediated by TCMs (107).

In parallel, network pharmacology and molecular docking combine database mining with computational simulation to predict active compounds, candidate targets, and related signaling pathways within multi-herb formulations (108). Liu et al. (58) collected prescriptions for infectious bronchitis from the TCM Inheritance Database and used network pharmacology to identify key components and targets of the modified MXSG formula, thereby guiding subsequent experimental validation. These in silico approaches are useful for exploring the multi-component, multi-target, and multi-pathway features of TCM formulations, but their predictions should be interpreted as hypothesis-generating and validated experimentally.

### 5.5 Emerging technologies for Candidate Identification, target validation, and standardization of anti-IBV herbal interventions

Emerging biotechnological and computational approaches may help address key challenges in anti-IBV herbal research, including compound identification, target validation, extract standardization, and scalable production.

For complex herbal extracts, AI-assisted approaches can prioritize candidate compounds and predict compound–target interactions (109, 110). However, these predictions require experimental confirmation in IBV-specific models. Structural biology can further validate whether predicted compound–target interactions are biologically meaningful, for example through structure-guided analysis of viral proteins such as the spike protein or PLpro (111). Synthetic biology and related techniques may improve batch consistency and scalability by enabling production of chemically defined active compounds (112–116). Collectively, the integration of AI-guided prediction, structural validation, and standardized manufacturing may facilitate the development of herbal interventions into reliable adjunctive tools for IBV control.

## 6 Discussion

Collectively, the evidence summarized in this review supports a predominantly host-directed but not exclusively host-directed mode of action underlying herbal interventions against IBV. Rather than relying on a single viral target or replication step, many herbal components influence host immune responses, stress-adaptive pathways, inflammatory homeostasis, oxidative balance, and cell-fate regulation. Through coordinated modulation of these processes, herbal medicines may restrict viral replication while simultaneously reducing virus-induced tissue injury (117, 118).

This multi-layered regulatory paradigm is particularly relevant for IBV, a highly mutable coronavirus for which vaccine-induced cross-protection is frequently incomplete (3–6). Current evidence indicates that herbal interventions may influence multiple stages of infection, including viral entry, replication, immune activation, and tissue protection. However, whether these effects confer protection against viral variation or immune escape remains experimentally unconfirmed. Therefore, herbal medicines should currently be investigated as complementary components of integrated IBV management strategies rather than replacements for established vaccination programs.

From a translational perspective, the host-oriented nature of many herbal antiviral mechanisms may offer potential advantages in poultry production. By modulating immune and inflammatory responses rather than exclusively targeting viral structures, herbal interventions may help preserve epithelial integrity and immune competence during infection. However, potential effects on viral evolution, including reduced selective pressure for mutation, remain experimentally unverified (119).

Beyond antiviral efficacy, successful veterinary application of herbal interventions requires comprehensive evaluation of practical and regulatory considerations. First, safety should be assessed at the effective antiviral dose rather than solely based on traditional usage history. Dose-dependent toxicity, tissue distribution, residue accumulation, and withdrawal periods must be established before application in food-producing animals. The absence of detectable toxicity in experimental models does not necessarily guarantee safety under prolonged administration or large-scale production conditions.

Second, the physicochemical stability and practical delivery of herbal preparations remain important challenges. Active constituents may vary depending on extraction procedures, storage conditions, feed composition, water quality, and environmental temperature. Therefore, formulation stability, palatability, bioavailability, and batch-to-batch consistency require standardized quality-control approaches before commercial use.

Third, interactions with existing poultry management practices should be systematically evaluated. Herbal formulations intended for use alongside vaccines or other veterinary treatments should be assessed for potential effects on vaccine-induced immunity, drug absorption, and therapeutic outcomes. In particular, combinations of herbal products with antibiotics should be considered within the framework of antimicrobial stewardship. Such combinations should be supported by evidence of clinical benefit and should not encourage unnecessary antibiotic use or substitute evidence-based antimicrobial management strategies.

Finally, regulatory approval and economic feasibility remain critical determinants of field adoption. Commercial application requires clearly defined active components, reproducible manufacturing processes, quality standards, regulatory compliance, and cost-effectiveness compared with existing control measures. Therefore, herbal interventions should currently be considered complementary tools within integrated IBV management programs rather than standalone replacements for vaccination, biosecurity, or veterinary therapeutics.

Despite the increasing number of studies investigating herbal interventions against IBV, the current evidence base remains heterogeneous and presents several limitations for comparison and clinical translation. Experimental models vary substantially in IBV strain, host system, cell type, animal age, infection route, viral dose, treatment timing, and outcome measurements, limiting direct comparison across studies.

In addition, many herbal extracts and compound formulations remain incompletely characterized. Differences in plant origin, extraction procedures, active constituent composition, and quality-control standards complicate the identification of responsible bioactive components and mechanisms. Moreover, several studies are based on limited experimental scales, and independent validation remains insufficient. Future investigations should prioritize standardized preparations, rigorous experimental designs, pharmacological characterization, and well-controlled poultry challenge models.

Not all herbal compounds or formulations discussed in this review have been tested in live chickens. Some interventions, such as APS, RIP, MXSG-related formulations, BQ oral liquid, SGDL, CTA, and PEO, have been evaluated in chicken embryo or live bird models, whereas others have mainly been assessed in cell culture systems, other coronavirus models, or network pharmacology analyses. A major limitation is that many reported anti-IBV activities of herbal medicines have been demonstrated primarily *in vitro* systems, including Vero, BHK, H1299, or CEK cells. Although these models are valuable for preliminary screening, dose–response evaluation, and mechanistic exploration, they do not fully reproduce the respiratory mucosal environment, tissue tropism, immune complexity, microbiota interactions, or pharmacokinetic processes of IBV infection in chickens. Therefore, reductions in viral RNA levels, cytopathic effects, or viral titers in cell culture should not be directly interpreted as equivalent to protective efficacy in poultry. Evidence derived from chicken embryos and live bird challenge models is more biologically relevant, but these models also vary in animal age, infection route, viral strain, inoculation dose, treatment timing, and outcome measurements, which complicates direct comparison across studies.

Another important concern is the heterogeneity of herbal interventions and the lack of standardization across studies. Existing studies differ substantially in source materials, extraction solvents, preparation procedures, active-component characterization, dosages, administration routes, and treatment schedules for single herbs, purified compounds, and compound formulations. For crude extracts and multi-herb prescriptions, incomplete chemical profiling and batch-to-batch variability limit reproducibility and make it difficult to identify the active constituents or dominant mechanisms responsible for the observed antiviral effects. In addition, many studies report antiviral activity without sufficient data on pharmacokinetics, bioavailability, tissue distribution, residues, or toxicity (120–123). These issues are particularly important for poultry production, where candidate interventions must remain stable in feed or drinking water, reach effective concentrations in respiratory or renal tissues, be compatible with vaccination programs and other veterinary drugs, and meet safety requirements for large-scale field application.

Future studies should therefore move beyond preliminary efficacy screening and adopt more rigorous and standardized evaluation frameworks. Priority should be given to chemically characterized formulations, clearly reported IBV strains and genotypes, standardized extraction and quality-control procedures, dose–response and time-of-addition analyses, pharmacokinetic and residue studies, toxicity assessment, and well-controlled animal trials under conditions that better approximate commercial poultry production. Such evidence will be essential for distinguishing broadly immunostimulatory or cytoprotective effects from true anti-IBV activity and for advancing herbal interventions from experimental candidates toward practical adjuncts for integrated IBV control.

## 7 Conclusion

Accumulating evidence suggests that TCM-derived interventions represent promising complementary candidates for IBV prevention and control. However, the current evidence remains heterogeneous, with substantial variation in experimental models, formulation standardization, mechanistic validation, and independent replication. Therefore, herbal interventions should currently be considered adjunctive candidates requiring further rigorous validation rather than established alternatives to vaccination and other control measures.

Nevertheless, further progress will require rigorous *in vivo* validation of key active constituents, deeper mechanistic clarification of compound prescriptions, and the development of standardized, safe, and practically applicable formulations for large-scale poultry production. Continued integration of modern pharmacology, omics-based technologies, and evidence-based veterinary evaluation will be essential for translating TCM-derived interventions from experimental candidates into reliable adjunctive tools for sustainable poultry health management and integrated IBV control.
